# New dating of the Matalascañas footprints provides new evidence of the Middle Pleistocene (MIS 9-8) hominin paleoecology in southern Europe

**DOI:** 10.1038/s41598-022-22524-2

**Published:** 2022-10-19

**Authors:** Eduardo Mayoral, Jérémy Duveau, Ana Santos, Antonio Rodríguez Ramírez, Juan A. Morales, Ricardo Díaz-Delgado, Jorge Rivera-Silva, Asier Gómez-Olivencia, Ignacio Díaz-Martínez

**Affiliations:** 1 Departamento de Ciencias de la Tierra, Facultad de Ciencias Experimentales, Campus de El Carmen, Universidad de Huelva, Huelva, Spain; 2 CCTH - Centro de Investigación Científico Tecnológico, Universidad de Huelva, Huelva, Spain; 3 DFG Center of Advanced Studies ‘Words, Bones, Genes, Tools’, Eberhard Karls University of Tübingen, Rümelinstrasse 23, 72070 Tübingen, Germany; 4 UMR 7194 Histoire Naturelle de L’Homme Préhistorique, CNRS, Muséum National d’Histoire Naturelle, Université Perpignan Via Domitia, Paris, France; 5 Departamento de Geología, Facultad de Geología, Campus de Llamaquique, Universidad de Oviedo, Oviedo, Spain; 6 Estación Biológica de Doñana-CSIC, Sevilla, Spain; 7 Centro de Investigación, Tecnología e Innovación (CITIUS), Universidad de Sevilla, Sevilla, Spain; 8 Dept. Geología, Facultad de Ciencia y Tecnología, Universidad del País Vasco/Euskal Herriko Unibertsitatea, UPV/EHU, Barrio Sarriena s/n, 48940 Leioa, Spain; 9 Sociedad de Ciencias Aranzadi, Zorroagagaina 11, 20014 Donostia-San Sebastián, Spain; 10 Centro Mixto UCM-ISCIII de Investigación Sobre Evolución y Comportamiento Humanos. Avda. Monforte de Lemos, 5 (Pabellón 14), 28029 Madrid, Spain; 11 Universidad Nacional de Río Negro-IIPG, General Roca, Río Negro Argentina; 12 Instituto de Investigación en Paleobiología Y Geología (IIPG), CONICET, General Roca, Río Negro Argentina

**Keywords:** Anthropology, Palaeontology

## Abstract

Hominin footprints were recently discovered at Matalascañas (Huelva; South of Iberian Peninsula). They were dated thanks to a previous study in deposits of the Asperillo cliff to 106 ± 19 ka, Upper Pleistocene, making Neandertals the most likely track-makers. In this paper, we report new Optically Stimulated Luminescence dating that places the hominin footprints surface in the range of 295.8 ± 17 ka (MIS 9-MIS 8 transition, Middle Pleistocene). This new age implies that the possible track-makers are individuals more likely from the Neandertal evolutionary lineage. Regardless of the taxon attributed to the Matalascañas footprints, they supplement the existing partial fossil record for the European Middle Pleistocene Hominins being notably the first palaeoanthropological evidence (hominin skeleton or footprints) from the MIS 9 and MIS 8 transition discovered in the Iberian Peninsula, a moment of climatic evolution from warm to cool. Thus, the Matalascañas footprints represent a crucial record for understanding human occupations in Europe in the Pleistocene.

## Introduction

The recently published discoveries and re-studies of Pleistocene hominin footprints in UK^1^, France^2,3^, Italy^4^, Spain^5,6^, and Greece^7^ have highlighted the crucial importance of the ichnological record in discussing Europe’s palaeoanthropological scenario.

While its use is common when studying fossil animal tracks, particularly dinosaurs, ichnotaxonomy, which refers to the definition of taxa based on the morphological features of tracks^8^, is rarely used for hominin footprints^9^. Only two ichnospecies have been defined from hominin footprints: *Praehominipes laetoliensis*^10^, for the famous Laetoli footprints, and *Hominipes modernus*^9^ defined from *Homo sapiens* footprints whose diagnostic characteristics (short digit impressions; a hallux impression about twice as long and wide as the other toes; the heel, lateral margin, ball and hallux impressions are the most strongly impressed) include all known footprints attributed to the genus *Homo*. Hominin footprints are in most cases attributed to taxa defined based on anatomical characteristics, which from a palaeoanthropological point of view allow a more precise classification than can be obtained through ichnotaxonomy. Attributing of hominin footprints to a particular taxon is generally done from indirect evidence. The association of footprints with archaeological^11,12^ or skeletal^12^ assemblages can reinforce a taxonomic assignation, although this occurs only in exceptional cases. Nevertheless, in most cases, the attribution is solely based on the chronological context (e.g.,^9^). This fact has highlighted the importance of using reliable dating tools to constrain this kind of hominin fossil remains chronologically.

**Figure 1:**
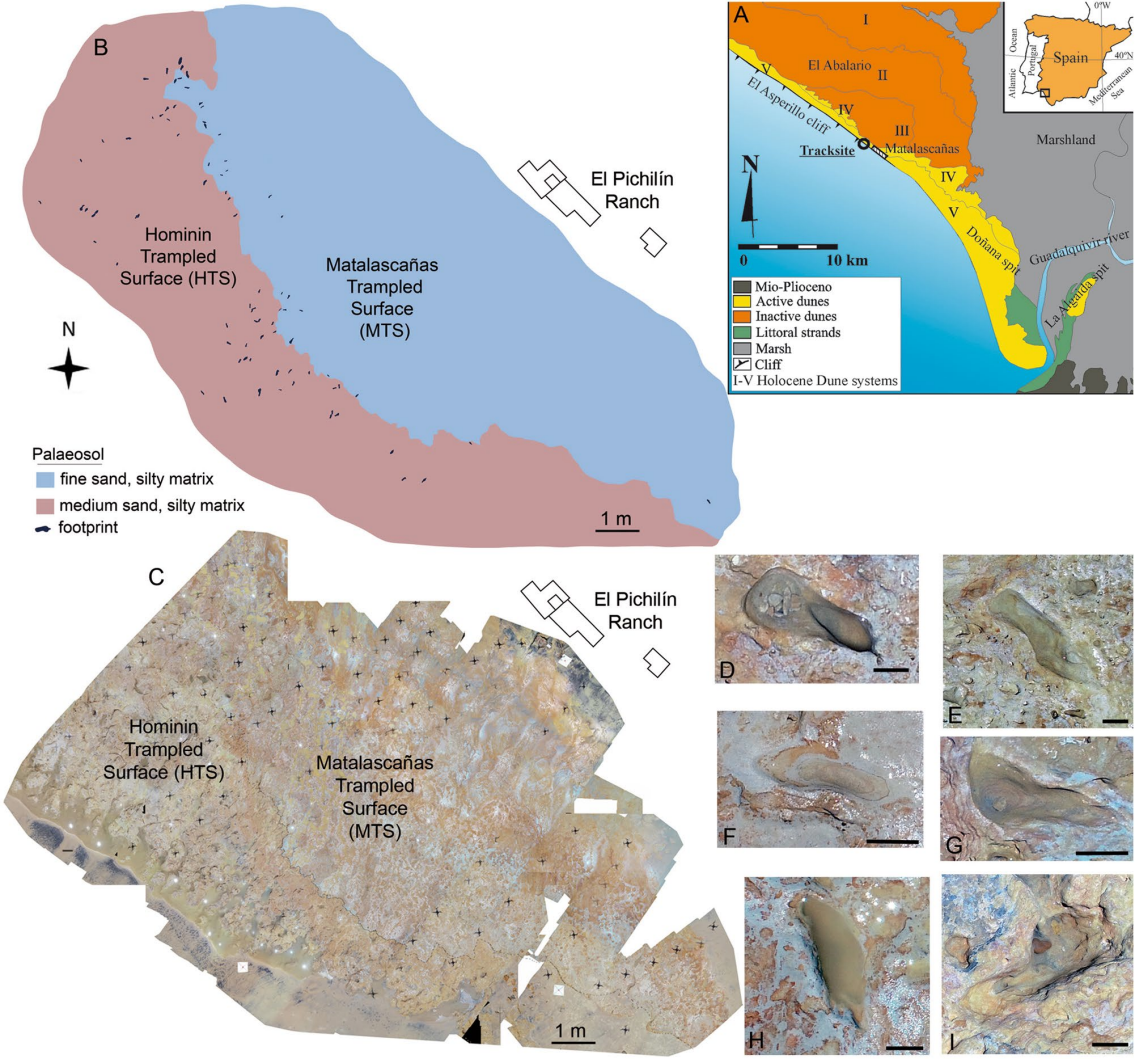
The Matalascañas site (El Pichilín Ranch area). (**A**) Geographical location and geological setting. (**B**) Mapping for the hominin footprints (HTS) and animal tracks (MTS) A and B from ^6^. (**C**) Aerial view of the drone flight. (**D**–**I**) Some relevant footprints from the outcrop. All scale bar: 5 cm. (**D**) M2020-02. (**E**) M2020-06. (**F**) M2020-05. (**G**) M2020-11. (**H**) M2020-74. (**I**) M2020-35. The map in (**B**) was created using Adobe Photoshop Versión: 9.0 (https://adobe-photoshop-9-cs2.en.lo4d.com/) based on the photogrammetric orthomosaic (**C**) produced using Pix4DMapper© (versión PIX 4D Cloud, https://www.pix4d.com/). (**C**) was obtained by a low-altitude programmed flight using an Unmanned Aerial Vehicle (UAV) on June the 6th, 2020, 12 AM UTC. A multirotor DJI Phantom 4 + with a 4 k 20 Mpix RGB CMOS camera was collected at an average height of 7 m above the ground photographs over the study area (564 m^2^).

Recently, a new track site with hominin footprints^6^ and tracks ascribed to the auroch *Bos primigenius*?, the red deer *Cervus elaphus*, the wild boar *Sus scrofa*, as well as Elephantidae (*Palaeoloxodon antiquus*), Canidae (*Canis lupus*), and waterbirds (geese, Anserinae, and waders, Charadrii)^13–15^ was studied in the Asperillo Cliff, close to Matalascañas (Doñana Shoreline, Spain, Fig. 1). An earlier dating of the aeolian unit located just above the palaeosol that preserves the footprints suggested an age before the date 106 ± 19 ka^16^. This chronological context made it possible to propose Neandertals as the possible producers of the hominin footprints. It also enabled a discussion of this palaeocommunity’s ecological role and evolutionary context in a favourable climatic moment such as the marine stage MIS 5^6,13,14^. However, in the development of our subsequent research and taking into account the importance of chronological data in palaeoanthropological studies, and with the aim of verifying and confirming the current dating, a new sampling was carried out in the Asperillo Cliff area, making particular emphasis on the levels of palaeosol and dunes directly related to the preservation of hominin tracks.

In this context, the objective of this work is multiple: (1) to provide a new chronology of the Matalascañas tracksite based on optically stimulated luminescence (OSL); (2) to highlight the palaeoanthropological importance of this record; and (3) to delve into the palaeoenvironmental and palaeogeographical scenario in which the footprints were impressed.

## Methods

### Grain size analysis

Grain-size analysis was developed in previously desiccated sediment samples by sieving, using a normalised base 2 logarithmic Udden-Wentworth scale. The percentage of each fraction of the sediment by weight was calculated for the total weight of the dry sample.

### Luminescence dating technique

Certain minerals, once buried, can store the charge released due to their exposure to ionizing radiation. This charge is stored in the crystal lattice. The luminescence dating technique is based on the measurement of the signal produced by quartz and feldspar minerals mainly, which can be obtained by thermal or by optical stimulation. In the first case, we consider the thermoluminescence technique, TL, while in the latter, we consider the optically stimulated luminescence, OSL. In both cases, the luminescence technique is used to determine the burial period (i.e. when the sediment was exposed to sunlight)^17,18^.

A charge is stored thanks to the impurities and missing atoms in the crystal lattice, which act as traps at certain energy levels in the forbidden bandgap (located between the valence band and the conduction band)^17,18^. Ionizing radiation produces a redistribution of charge, and a portion of free electrons are trapped in the impurities of the crystal lattice^17,18^.

The time the crystal has received radiation after being buried makes a unique determination of the amount of trapped charge. Luminescence can result from the stimulation of specific energy. Electrons escape into the conduction band when energy greater than the energy gap between the trap and the band is applied. Some of the electrons could become trapped again, and a small percentage of the remaining electrons in the RC recombination centres would recombine with the trapped holes. These recombination reactions can be radiative, causing luminescence in some cases^17,18^. These metastable energy levels can be depleted by daylight, so the crystal only accumulates the trapped charge created by ionizing radiation when it is buried and protected from light. Using an appropriate optical stimulation wavelength, the OSL signal can be measured in the laboratory. Blue (470 nm) or green (530 nm) stimulation is used for quartz stimulation. After this stimulation, the natural luminescence signal of a sample, corresponding to the dose received by the sample during its burial time, is evaluated, and the equivalent dose is calculated^18,19^.

The dose per unit time that a mineral receives in nature (known as dose rate) incorporates all the ionizing radiation from all sources, including cosmic rays, which have been corrected for burial depth, and the disintegration of radioactive nuclides found nearby. The final dose rate received is also influenced by the water content of the environment, in which water attenuates radiation^18,19^.

The age of a particular material can then be estimated from the expression.

$$Age\, \left(ka\right)=\frac{Equivalent\,Dose (Gy) \, }{Dose\,rate (\frac{Gy}{ka})}$$

where Gy denotes the unit of dose, Gray, equivalent to 1 Joule of absorbed energy per kg of matter, and ka is one thousand years^17^.

### Sampling method

Four samples for grain-size analysis were obtained from the clean outcrop surface using a small plastic spade. These samples were conserved in plastic bags identified with their acronyms.

The sampling for OSL chronology was carried out by obtaining manual cores by pounding in an opaque PVC pipe of 60mm diameter and a wall of 3 mm. After the core extraction, the ends were occluded using outer plugs, opaque, and fixed using duct tape. Each core was labelled on the PVC pipe with its acronyms and polarity.

### Luminescence dating

**Figure 2:**
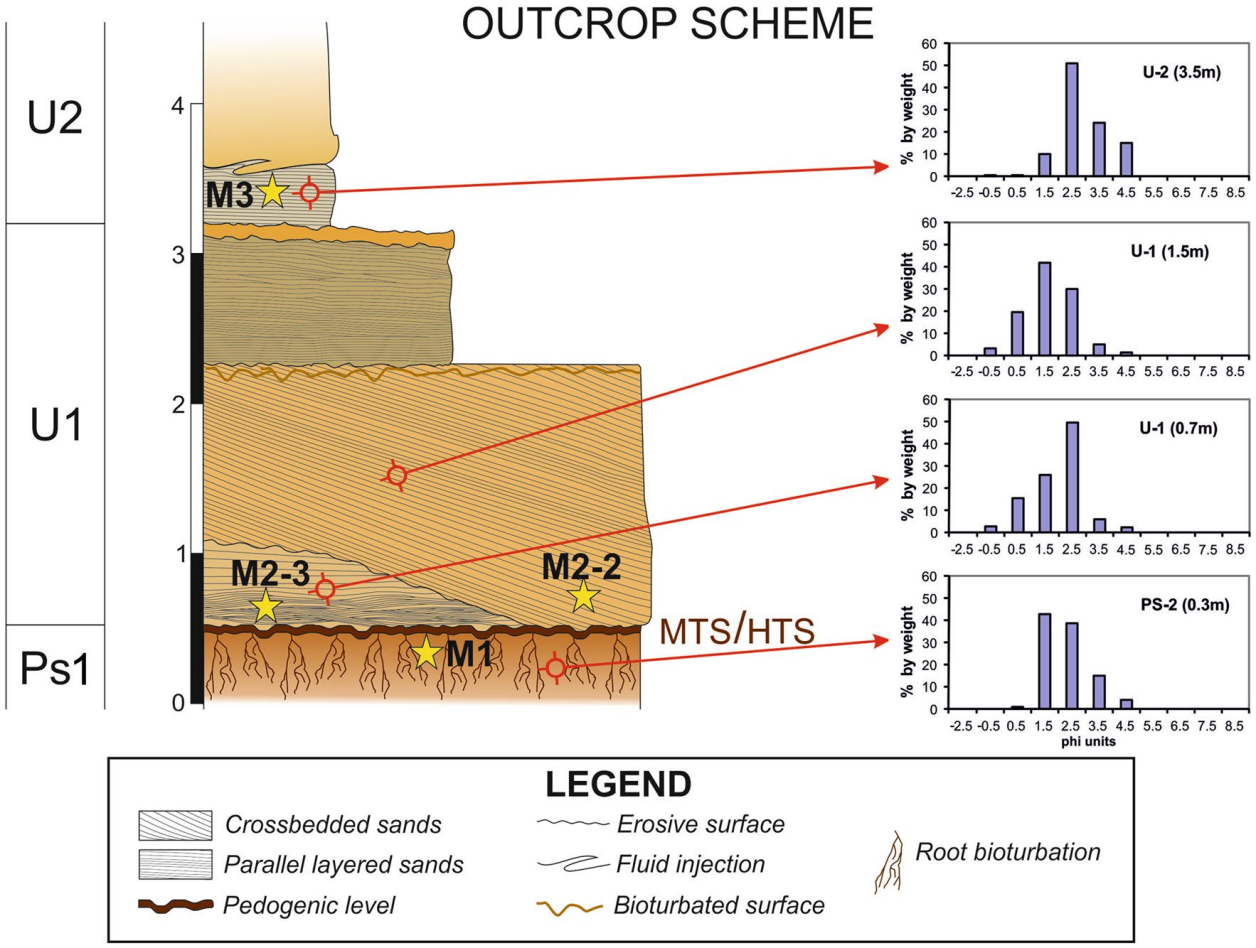
Sedimentary log of the footprints site in Matalascañas. The samples were obtained for dating (M1 to M3) and sediment grain-size histograms for the analysed samples. Units according to^16^. MTS: Matalascañas Trampled Surface^13^. HTS: Hominin Trampled Surface^6^.

A total of four sedimentary units from the Asperillo cliff were selected for optically stimulated luminescence (OSL) dating (Fig. 2). They were all collected using opaque tubes, avoiding the exposure of the sediment to daylight and were then treated in the laboratory under controlled light conditions. Quartz grains of sizes 180–250 µm were extracted from each sample using standard procedures^20^.

### Sample preparation procedure

Samples were wet sieved to obtain fractions of 90–180 μm and 180‐250 μm. The latter fraction (180–250 μm) was used in this study. These fractions were chemically treated to isolate quartz grains. First, samples were treated with 10% HCl until carbonates were dissolved, and H_2_O_2_ was used to eliminate the organic matter. Next, two density separations were performed using sodium polytungstate to isolate quartz from feldspar and heavier minerals. A solution of 40% HF was then used to eliminate feldspar residuals and etch the quartz outer layer, which would have been affected by alpha radiation. Finally, the resulting quartz grains were dried and sieved, and the fractions of 180‐250 μm were chosen to carry out the measurements.

### SAR procedure

Samples were measured using the SAR procedure^21^. In the SAR procedure (Single Aliquot Regenerative dose), the sample is divided into aliquots which are measured individually. SAR includes several measurement cycles. In the first cycle, the native OSL signal obtained from the sample (L_N_) is measured (natural dose). In the following cycles, the sample is irradiated with known doses, provided with a calibrated radiation source, and measured by the obtained OSL signals (L_1_, L_2_,…). Before carrying out the measurement, the aliquot is preheated to a specific temperature to eliminate the electrons from the most superficial traps so that the OSL signal obtained is more stable and truly corresponds to the burial period.

To determine the pre-heat temperature, a previous study was carried out with bleached aliquots, subsequently irradiated with a known dose, similar to the natural dose. Then, the given/recovered dose relationship is determined for each temperature. Finally, the temperature for which the best value is obtained (closest to the unit with the least uncertainty) is chosen as the pre-heat temperature for the study. In our case, pre-heat temperatures between 220 °C and 240 °C have been considered.

To determine the sensitivity of quartz grains, the sample is irradiated with a constant reference dose, called the test dose. The test dose signal is measured at each cycle (T_N_, T_1_, T_2_,…) to correct possible luminescence changes in the measurement cycle, mainly due to pre-heat treatment.

From the measurements made, the equivalent dose D_e_ is obtained. De is equivalent to the total dose that the sample has received during the burial period. To determine D_e_, a curve is interpolated. In this curve, the signals obtained in each measurement cycle are divided by the test doses (L_N_/T_N_, T_1_/T_1_, L_2_/T_2_, ...) to correct the quartz sensitivity effect. The SAR protocol has been accepted to provide the most accurate De estimates because it is designed to correct for such changes in quartz sensitivity.

### OSL measurement and analysis

OSL has been measured using Risø OSL/TL readers (TL-DA 20) with a calibrated ^90^Sr/^90^Y beta source delivering ~0.10 Gy/s at the sample disc location. The OSL signal from 30 to 60 small quartz multi-grain aliquots (1 and 2 mm diameter) was measured from each aliquot. Thirty aliquots were measured by default. Measurements were extended to 60 aliquots in cases where the overdispersion was higher than 35%.

#### Preheat dose recovery test

**Figure 3:**
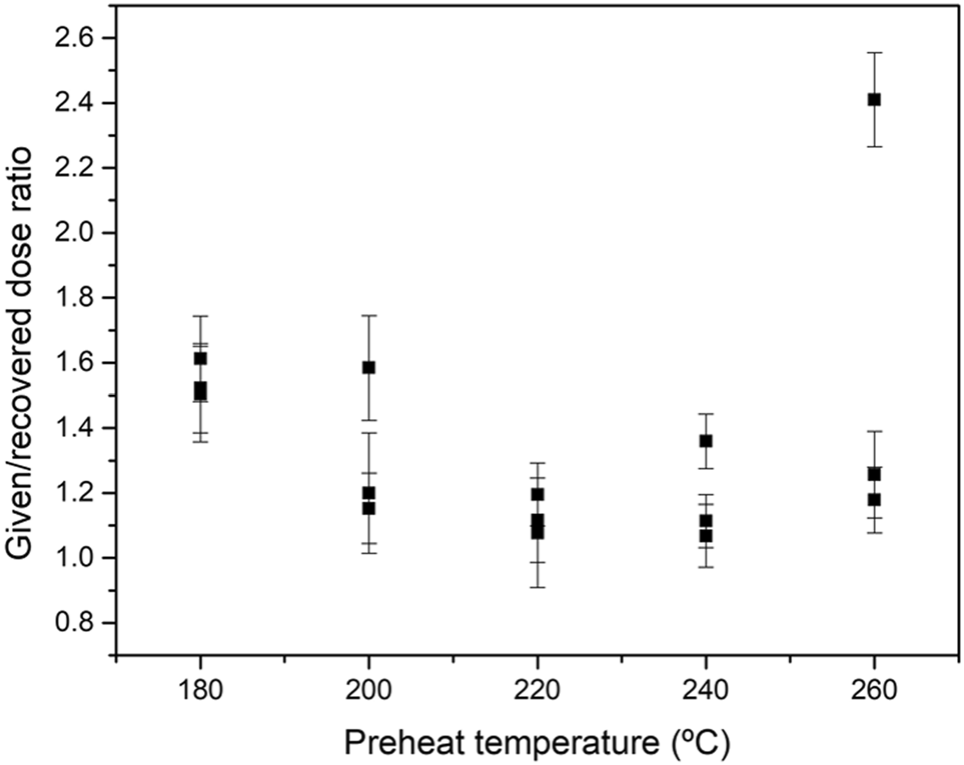
Preheat dose recovery test results for sample M1.

A pre-heat temperature of 220 °C was considered for the OSL measurements. A pre-heat dose recovery test was carried out for sample M1 to determine this temperature. Fifteen aliquots were bleached using a daylight simulator for 5 hours and subsequently irradiated with 200.2 Gy, previously determined by a dose range test. Five sets of 3 aliquots each were preheated at 5 different temperatures (180 °C, 200 °C, 220 °C, 240 °C and 260 °C). Then, the given/recovered dose ratio was determined for each preheat temperature. The best averaged given/recovered dose ratio was obtained for 220 °C (Fig. 3).

#### OSL measurements

**Figure 4:**
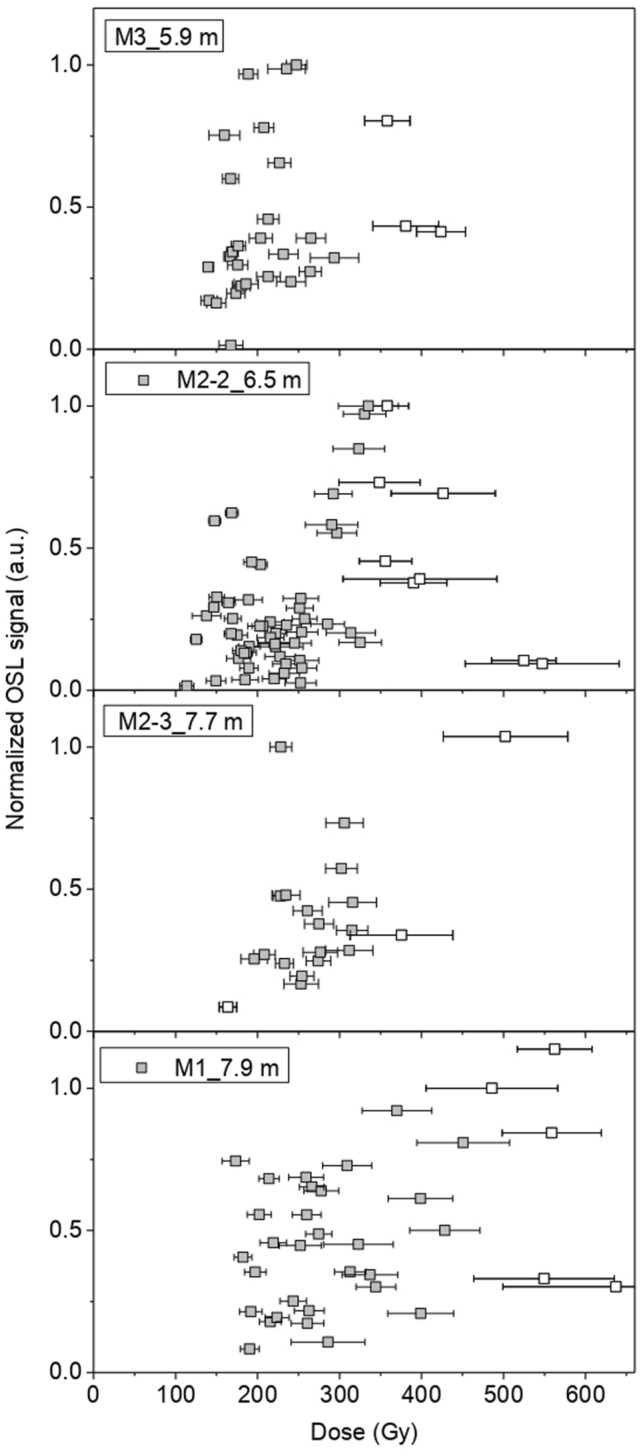
Dose distributions derived from OSL measurements. The normalised OSL signal is plotted as a function of the individual dose values.

**Figure 5:**
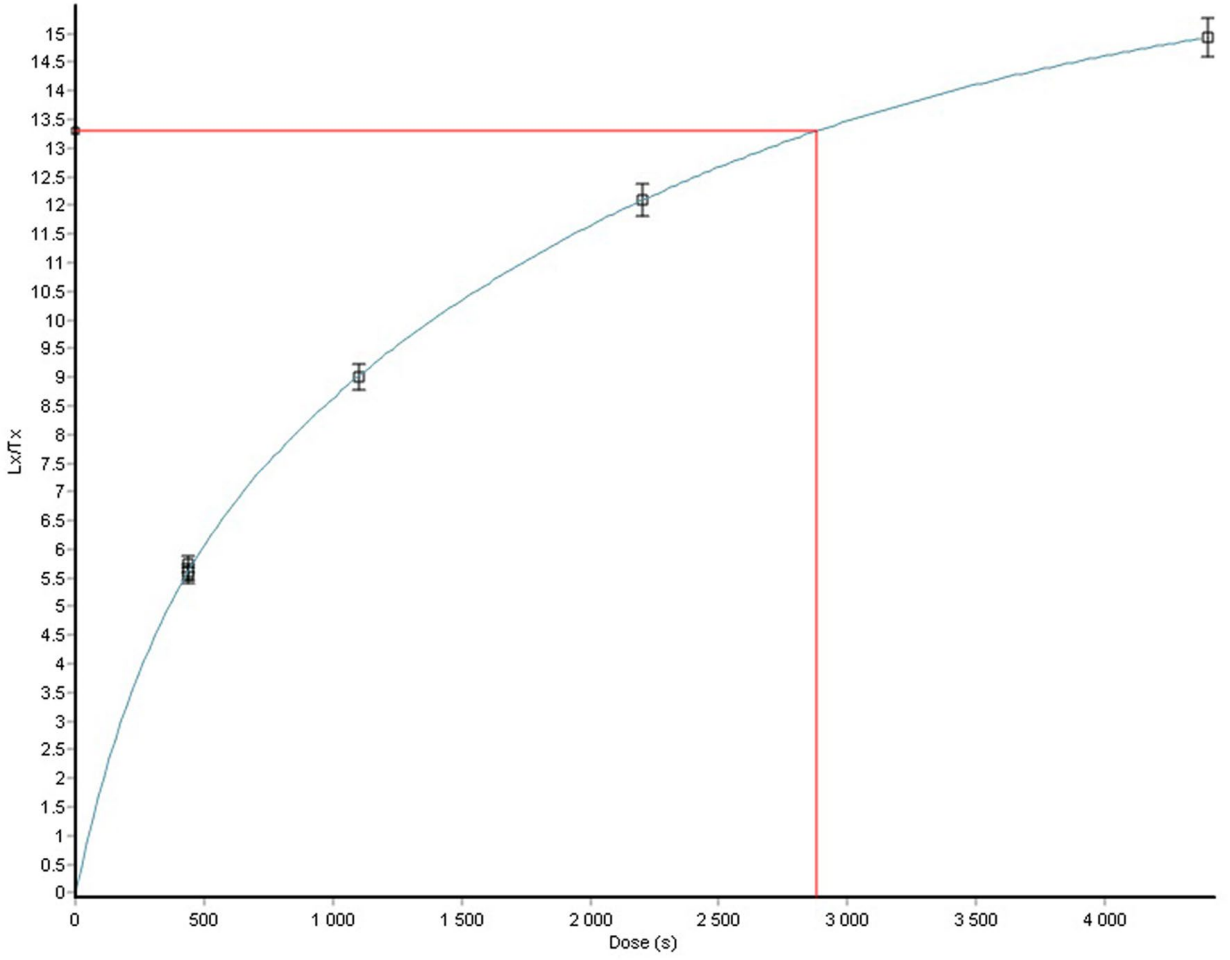
Dose–response curve representative of the measurements performed.

**Figure 6:**
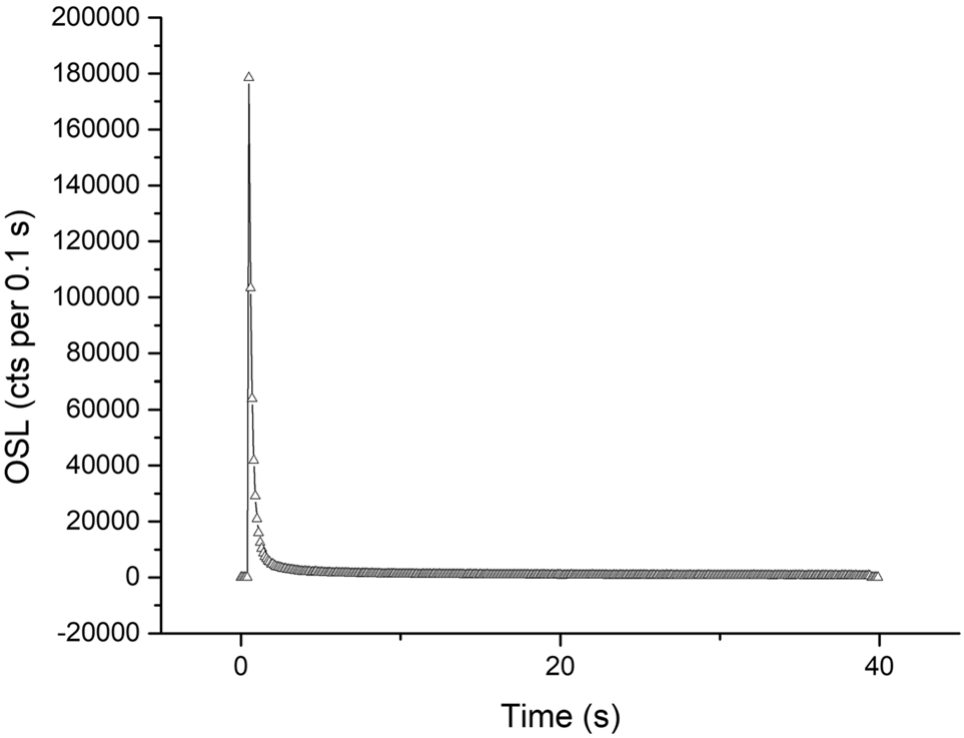
OSL decay curve representative of the measurements performed.

Table 1 shows the SAR protocol used in this study. The OSL signal was measured for 40 s at 0.1 s per data point, giving 400 data points (Fig. 4). OSL signal was measured using a blue LED as the light source. The reading temperature for OSL was 125 °C. The fast component dominated the signal, and the first 5 data points (0.5 s) were considered for measurement. The background was calculated from the final part of the spectrum, taking 50 data points, and subtracted from the measured signal. Feldspar contamination was also tested using IR simulation. No detectable IRSL signal was observed. Figures 5 and 6 have been chosen as representatives of the OSL measurement process. They show the OSL decay and dose-response curves, obtained from sample M1. Supplementary material (Annex S1, S2, S3, S4) contains examples of these curves for all the samples measured.

**Table 1:** SAR protocol.

Step	Treatment	Measurement
1	Give regenerative dose	–
2	Preheat (220 °C, 10 s)	–
3	OSL (Blue at 125 °C, 40 s)	Lx
4	Give test dose	–
5	Cutheat (200 °C, 10 s)	–
6	OSL (Blue at 125 °C, 40 s)	Tx
7	Cleanout (280 °C, 100 s)	–
8	Return to step 1	–

Derived dose distributions have been reduced by removing individual outliers, identified as those values out of 1.5 times the interquartile range. The resulting populations are normally distributed with overdispersion values <25% (Fig. 4). The Central Age Model (CAM^22^) has been applied to calculate the equivalent dose (i.e. the accumulated dose due to the ionising radiation received by the quartz grains over the period they have been buried) of each sample.

### Dose rate calculations

Total dose rates have been calculated from the activity concentrations of ^40^K, T^232^Th and ^238^U, measured by high-resolution low-level gamma spectrometry (Table 2). These measurements were made using high-purity Germanium semiconductor detectors, HPGe, cryogenic cooled and surrounded by passive and active shielding to avoid external influence. The contribution of the cosmic radiation to the total dose rate has been calculated as a function of the latitude, altitude, burial depth and the average overburden density, based on^23^.

**Table 2:** Summary of the results and parameters used for OSL dating. Lab codes from the Centro Nacional de Investigación sobre la Evolución Humana—CENIEH (LM) and University of Seville (RDI) laboratories are also indicated.

Sample	Lab code	No. of aliquots measured (selected in brackets)	Altitude (m)	Overburden density (g/cm^3^)	Depth (m)	Water content (%)	^40^K (Bq/kg)	^232^Th (Bq/kg)	^238^U (Bq/kg)	^40^K (%)	^232^Th (ppm)	^238^U (ppm)	Water corrected beta dose rate (Gy/ka)	Water corrected gamma dose rate (Gy/ka)	Cosmic dose rate (Gy/ka)	Overdispersion (%)	Dose rate (Gy/ka)	Burial dose (Gy)	Age (ka)
M3	LM20139-07	48 (30)	3.5	1.8	5.9	15 ± 5	231 ± 10	5.11 ± 0.38	3.8 ± 1.2	0.682 ± 0.059	1.26 ± 0.19	0.29 ± 0.19	0.64 ± 0.04	0.22 ± 0.02	0.104 ± 0.010	19	0.97 ± 0.05	197.3 ± 7.6	203.8 ± 12.7
M2-2	LM20139-05	68 (52)	0.7	1.8	6.5	20 ± 5	263 ± 12	8.07 ± 0.38	5.6 ± 1.3	0.776 ± 0.071	1.99 ± 0.19	0.43 ± 0.20	0.54 ± 0.03	0.28 ± 0.02	0.098 ± 0.010	24	0.91 ± 0.04	211.2 ± 7.4	232.8 ± 13.1
M2-3	LM20139-06	24 (18)	0.7	1.8	7.7	25 ± 5	239 ± 11	6.88 ± 0.48	4.7 ± 1.5	0.705 ± 0.032	1.70 ± 0.24	0.36 ± 0.23	0.63 ± 0.04	0.23 ± 0.02	0.087 ± 0.009	28	0.95 ± 0.04	259.3 ± 8.8	274.0 ± 15.8
M1	RDI-4233	48 (27)	0.3	1.8	7.9	25 ± 5	259 ± 5	11.9 ± 0.35	6.8 ± 0.7	0.764 ± 0.016	2.93 ± 0.09	0.53 ± 0.06	0.53 ± 0.03	0.30 ± 0.02	0.085 ± 0.008	25	0.91 ± 0.03	269.9 ± 13	295.8 ± 17.8

Dose rate calculations have been determined using the Dose Rate and Age Calculator (DRAC)^24^. DRAC is a web-based program developed for environmental radiation dose rate calculation for trapped charge dating applications. The program enables published attenuation and conversion factors to make accurate and reproducible dose rate calculations. Water content (Table 2) has been considered representative of the burial period. Conversion factors have been used according to^25^. The contribution of the cosmic radiation to the total dose rate has been calculated as a function of the latitude, altitude, burial depth and the average overburden density, based on^23^ (Table 2). Alpha grain-size attenuation was determined according to^26^. According to^25^, Beta grain-size attenuation was determined. The minimum and maximum etch depths were 8 and 10 microns, respectively. Beta etch depth attenuation factor was used according to^27^.

## Results and discussion

### Sedimentology and geochronology

**Figure 7:**
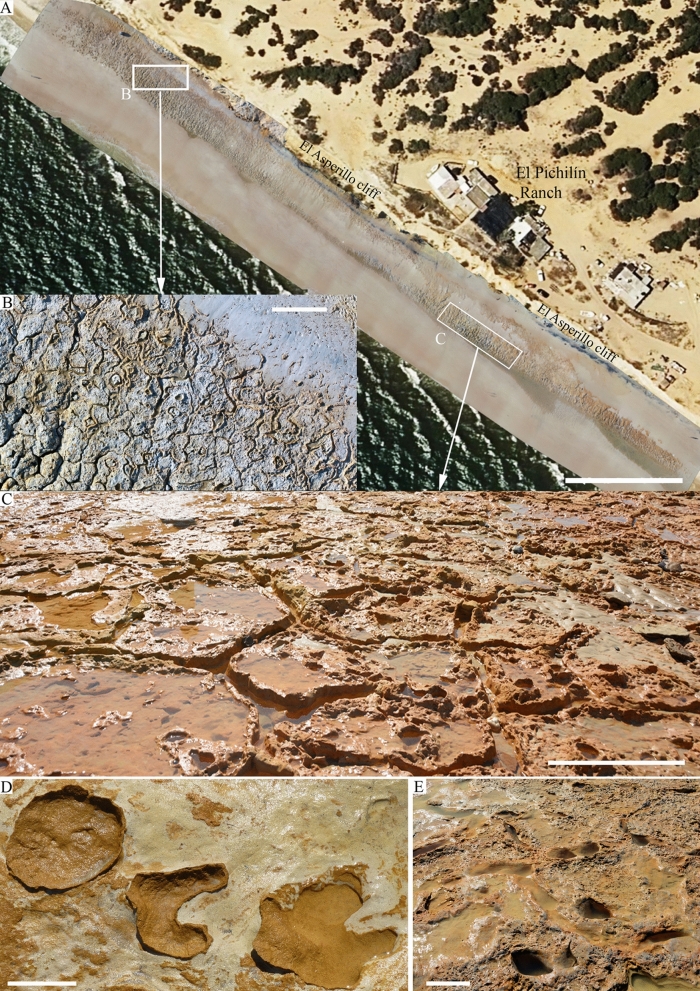
Current intertidal zone in the area surrounding El Pichilín, Castilla beach, Matalascañas. (**A**) General view of the cut-wave platform. Scale bar: 50 m. The polygonal mats with upturned margins of individual polygons show an extensive open cracks network resulting in the saucer-shaped polygons. Locally, subcircular openings with upturned and curled margins of the thin mat can also be seen. (**B**) Detail of the NW sector. Scale bar: 2 m. (**C**) Detail of the SE sector. Scale bar: 1 m. (**D**) *Cervipeda* and undetermined tracks were preserving remains of the microbial mat trapped under their footprints. Scale bar: 8 cm. (**E**) Hominin footprints preserved on the polygonal mats. Scale bar: 20 cm. The photogrammetric orthomosaic (**A**) was produced using Pix4DMapper© (versión PIX 4D Cloud, https://www.pix4d.com/). (**A**) was obtained by a low-altitude programmed flight using an Unmanned Aerial Vehicle (UAV) on June the 12^ht^, 2020, 12 AM UTC. Photograph was collected by a multirotor DJI Phantom 4 + with a 4 k 20 Mpix RGB CMOS camera at an average height of 50 m above the ground.

The stratigraphic sequence of El Asperillo Cliff was previously defined by previous authors^16^ and consists of a series of Pleistocene units interpreted by these authors as aeolian bodies. These units are named starting at the base: U1, U2 and U3. Each unit is separated from the others by bioturbated fine-grained beds, interpreted as pedogenic levels, paleosols or lacustrine beds. These palaeosols are ussually rich in clays and organic matter, but can also have a ferruginous composition. The lowermost pedogenic level PS1^28^, located immediately under U1, is characterised by yellowish-reddish mottled colours and an extensive network of polygonal mats of at least 300 m in length, which is the maximum surface area exposed so far (Fig. 7) which preserves both hominin fossil footprints^6^ and other associated vertebrate tracks^13–15^. Medium-fine sands form PS1 with a silty matrix (sample PS-2/0.3 m) displaying a strong vertically elongated arrangement of mottling, suggesting a genetic relationship with plant-root activity^28^. The top of this level presents a significant irregular surface with a millimetre-scale ferruginous crust which imprints the tracks. This surface slightly slopes to the SW. PS1 was OSL-dated (Sample M1) and yielded an age of 295.8 ± 17.8 ka (Table 2).

Unit U1 consists of three different sandy bodies (Fig. 2). The lowest is composed of moderately-sorted fine sands (sample U-1/0.7 m, dating sample M2-3, age 274.0 ± 15.8 ka, Table 2). The internal ordering of this body presents a base constituted by sets of hummocky cross lamination that evolve to parallel lamination slightly tilted to the NE. This first body results from strong water currents as indicated by the presence of hummocky cross-bedding. The second body of U1, constituted by moderately poorly-sorted medium sands (Sample U1/1.5m, dating sample M2-2, age 232.8 ± 13.1 ka, Table 2), presents an erosive base that cuts the lower body neatly and a clear planar crossbedding tilted with a NE vergence. A new erosive and bioturbated surface cuts the top of this crossbedded set. This second body was deposited under landward flows. The presence of land-inclined metric planar cross-bedding suggests being originated from a migrating aeolian dune. The third body of U1 is a metric level of parallel laminated well-sorted medium sands. This level is the top of U1 and could represent a set of parallel lamination similar to the deposited in an aeolian plane sheet. The entire sequence of U1 is similar to the typically developed in a dune field. At the top of the sequence of U1, a new pedogenic level with less entity than PS1 is present. This is a 10 cm sandy mud level with traces of plant bioturbation. The interpretation of U1 agrees with the vision of previous authors^16^, who suggest a dune origin for these facies.

From unit U2, only the base was analysed in this study. This basal body consists of a metric set of convolute laminated fine sands with significant amounts of very fine sands and silts (Sample U2/3.5m, dating sample M3, age 203.8 ± 12.7 ka, Table 2). The top of this body presents an irregular surface, including flame and charge structures, indicating fluidised sediments injection into the sandy upper body. The presence of these structures indicates an apparent water saturation of sediments during an earthquake. The upper bodies of U2 were not analysed, but they are described in the previous papers^6^ as well-sorted fine sands with complex curved-base cross-stratification. These upper bodies of U2 appear to be aeolian dunes as interpreted by the previous authors^16^.

### Palaeoenvironmental framework

The new chronology allows us to place the surface with hominin and other vertebrate footprints in the transition between MIS 9 and MIS 8 (295.8 ± 17.8 ka). Since then and until the Holocene, several stadials and interstadial periods have followed one another. Cold and dry climates characterise these stadials in the centre of the Iberian Peninsula and especially in northern Europe. However, the coastal areas of the Gulf of Cadiz served as reservoirs of thermophilic plant communities in these cold periods, favouring the settlement of human populations^29^. This would indicate a more temperate and humid climate, with high phreatic levels, abundant vegetation and edaphic and/or lagoon development. This new scenario contrasts with the previous geodynamic and chronological paradigm established^16,28^, which determines a wetter landscape in the interstadial periods, with edaphic development, and a drier landscape in the stadial periods, where dune dynamics dominate. This does not agree with the sedimentary record studied and the new chronology proposed in the present paper.

The sedimentary sequence outcropping at the Asperillo cliff shows a succession of beach-dune episodes and edaphic and/or pond surfaces, with sedimentary facies associated with the dynamics of the pond and tributary streams when aeolian activity ceased. The series above the Hominin Trampled Surface (HTS) starts with a dune deposit during MIS 8, with a direction of advance towards the E and ESE. During the last phases of MIS 9 in transit to MIS 8, the sea level would be, according to the composite sea-level curve for the Pleistocene epoch^30^, about 60 m below the present sea level, which would imply a shoreline position 20–25 km away from the present position. In this scenario, a very extensive coastal plain would develop with large flooded areas in shallow and hypersaline lacustrine environments. In this sense^28^, believe that from a stratigraphic point of view, the palaeosol PS1 represents the surface of an ancient fluvio-deltaic coastal plain so that the exposed palaeosols correspond to the same pedogenic cycle, which would have acted in the zone prior to c. 100 ka BP during the Middle-Late Pleistocene transition. This environment could be covered by water during humid seasons and totally or partially exposed during dry seasons. As a result, polygonal mats characterized by wide cracks with upturned margins producing individual saucer-shaped polygons were formed (Fig. 7A–C) and trampled, both by hominins and the rest of the vertebrate faunas (Fig. 7D,E). Currently, such networks of polygonal mats are found in extensive areas of tidal flats in both hot desert climates^31,32^ and tropical^33^ climates.

**Figure 8:**
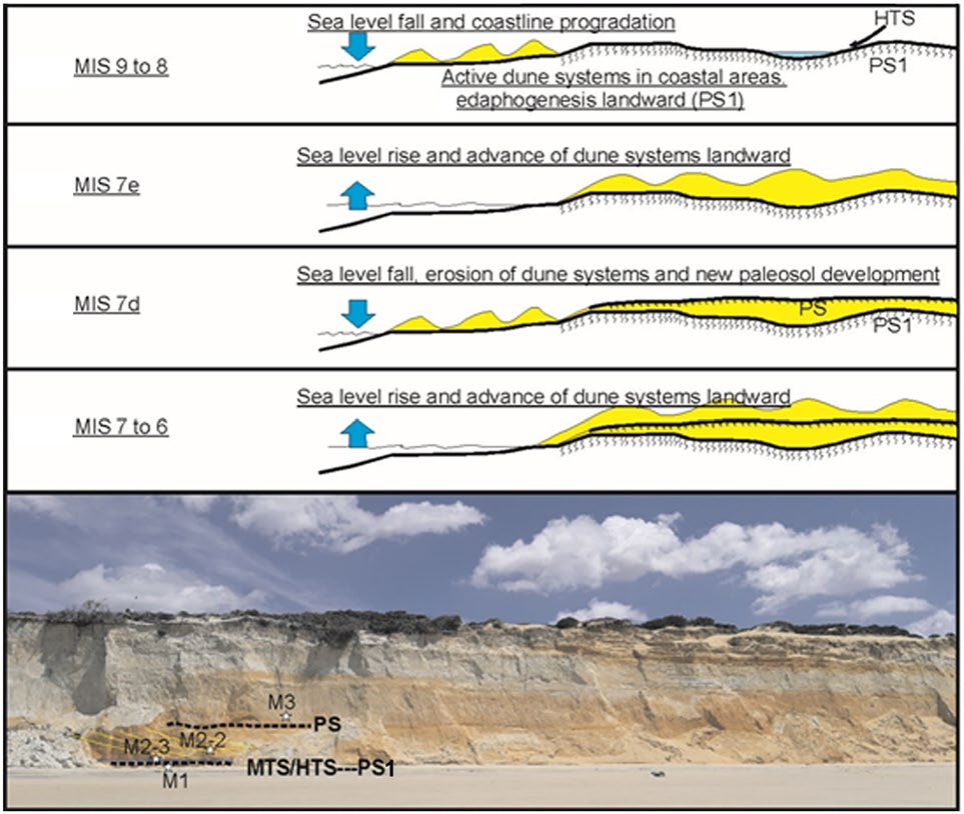
Proposed palaeoenvironmental context of Matalascañas and the evolution of the dune deposits about the rise and fall of sea level are presented.

In the non-flooded areas of this broad coastal plain hydromorphic processes related to root activity developed, constituting more or less extensive vegetated zones. The pedofeatures of the PS1 indicate, according to^28^, a long period of soil development related to a well-developed plant cover living on mature soils. Around these areas would expand a significant development of dune systems moving landward from the coast, similar to during the Holocene climatic optimum^16^. High sea positions and interstadial periods would favour this development of dune systems (Fig. 8).

As the sea retreated during MIS 8, the coastal plain would continue to expand by several tens of kilometres. The most active dune sequence would accompany this marine retreat, confining itself to the outermost coastal area. In a cooler and wetter climatic context, the former MIS 9 dunes would remain inland and progressively colonised by vegetation. The wind systems would be stabilised by vegetation, with edaphic development and pond proliferation in the topographically lower areas, with associated sedimentary facies due to surface water dynamics. We must not forget the hydrogeological conditions of the sandy substratum favouring the contribution of phreatic water to the wetland mosaic.

The series above the Hominin Trampled Surface (HTS) starts with a stream deposit during MIS 8. The sand bodies display a direction of advance towards the E and ESE. During MIS 7, the new sea rise would result in a further beach-dune advance landward, fossilising the soil and lagoon surfaces, in the context of a more significant aeolian activity, which would be interrupted by the wetter episode of MIS 7d and the subsequent drop in sea level. After that, the warmer phase of MIS 7a, b and c and the consequent rise in sea level would be accompanied by a new episode of aeolian activity, which ended during MIS 6.

In the El Asperillo Cliff area, the study of pollen levels corresponding to the cold periods of the Pleistocene would indicate a wetter and colder climate than the current one, with the presence of palaeochannels freshwater environments^34^. The pollen record from El Asperillo in the initial phases of the last glaciation reinforces the refugial character of these areas for Euro-Siberian and Atlantic taxa during the cold stages^34,35^. Similarly, in other areas of the eastern Iberian Peninsula, the influence of the Mediterranean Sea may have played an important role in attenuating the extent of this climatic deterioration compared to other inland regions of the Iberian Peninsula^36^. Thus, coastal areas must have reflected a landscape that offered a broad and constant spectrum of possibilities for past human populations.

### Taxonomic and palaeoanthropological implications

**Figure 9:**
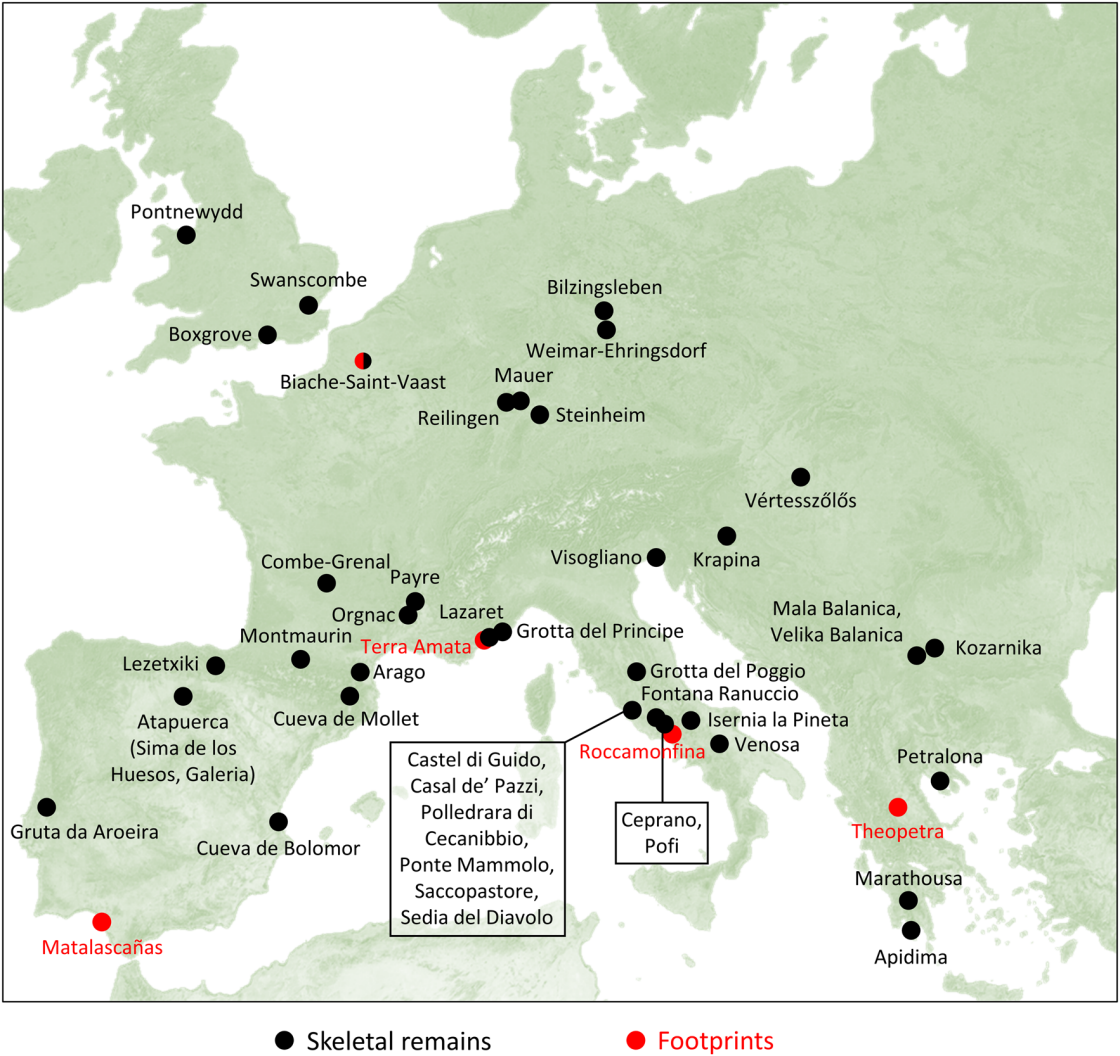
Geographical distribution of the main palaeoanthropological sites for the European Middle Pleistocene. Only those containing skeletal and ichnological assemblages are represented. The map was created from a free template (https://commons.wikimedia.org/wiki/File:Europe_satellite_image_location_map.jpg). The legends were added using Inkscape (v.1.0.2, https://inkscape.org/).

The new chronology at Matalascañas indicates that the human footprints would have been made nearly 200,000 years prior to the time established in their initial study^6^. This chronological shift places these new footprints during the Middle Pleistocene, specifically at the transition between MIS 9 and MIS 8. This new context is relevant since it provides information about the Middle Pleistocene fossils, a fragmentary and geographically heterogeneous record (Fig. 9), attributed to taxa belonging to the Neandertal lineage (i.e. to *Homo neanderthalensis* or *Homo heidelbergensis* s.l.). Footprints are even more scarce than skeletal remains in the entire European Middle Pleistocene since only four sites have delivered footprints from this period: Terra Amata^37^ and Roccamonfina^4^, dated to 380,000 and 345,000 years ago, whose footprints were attributed to *Homo heidelbergensis*, and the sites of Biache-Vaast^12^ and Theopetra^7^, dated to 236,000 and 130,000 years ago, whose footprints were attributed to *Homo neanderthalensis*. The dimensions of these Middle Pleistocene footprints are known except for the one from Biache-Saint-Vaast. The length ranges of the Matalascañas footprints (14–29 cm^6^ include those of the other sites. The two footprints measured at Theopetra (14–15 cm^7^ are close to the shortest Matalascañas footprints in contrast to the footprints from the Roccamonfina trackways (24–27 cm^4^ and the Terra Amata footprint (24 cm^37^). In particular, evidence of human occurrences dating to MIS 9 and MIS 8 is extremely rare^38^. They are mainly known from archaeological material (e.g. lithic industry, hearths, faunal remains), including some occurrences in the Iberian Peninsula^39^, such as at Bolomor Cave^40^ or Loma de los Huesos^41^ (Supplementary Table S1 online). European palaeoanthropological record (hominin skeletons and footprints) from these stages is much more scarce than archaeological material. Except for the Matalascañas footprints, no other hominin footprints from MIS 9-MIS 8 are known (Supplementary Table S2 online).

On the other hand, the number of paleoanthropological remains is reduced for the MIS 8 (Supplementary Table S1 online). Within the Iberian Peninsula, the footprints from Matalascañas are, to our knowledge, the only palaeoanthropological remains dating from MIS 9 or MIS 8 that have been published.

The taxonomic attribution of the Matalascañas footprints has to be discussed in light of the new dating. Indeed, in their initial study, they were attributed to Neandertals based on the first dating estimated at 106 ± 19 ka by^16^ in^6^.

This method of taxonomic attribution based on chronological context is common for hominin footprints. Indeed, ichnotaxonomy, usually used in the study of dinosaur tracks, is very little for hominin footprints. The ichnotaxon *Hominipes modernus*^9^ can include all known footprints attributed to the genus *Homo* by its diagnostic characteristics: from Ileret footprints potentially made by *Homo ergaster* or *Homo erectus l.s.*^42^ to the most recent *Homo sapiens* footprints. This inability to differentiate between human species constitutes one of the limitations of ichnotaxonomy in palaeoanthropology, at least for the most recent species that are best known from skeletal remains.

Furthermore, the taxonomic attribution of hominin footprints can be supported by geometric morphometry studies highlighting the anatomical features of the track-maker foot^2,43^. For instance, the attribution of footprints discovered at Le Rozel (80 ka, France) to Neandertals was supported by morphological differences between these footprints and those made by *H. sapiens*. The Le Rozel footprints were wider, especially at the midfoot level, reflecting a more robust foot and a less pronounced longitudinal arch^2^, anatomical characteristics known in the Neandertals fossil record^44,45^. However, such anatomical inferences are a challenging task, as the morphology of the footprint does not only result from the anatomy of the foot but also other factors such as biomechanical characteristics, the type of substrate or taphonomy. Therefore, the studied footprints must be well preserved and reflect several anatomical features (toe impression arch), which is rarely the case in loose substrates, such as the Matalascañas, where morphological variation is very important^46^.

If anatomical features can be obtained from the Matalascañas footprints, it will be necessary to compare them to the known Middle Pleistocene skeletal record to support a taxonomic assignment. Unfortunately, this record is scarce and heterogeneously distributed, spatially and temporally (Supplementary Table S1 and S2 online). Almost all of the known foot fossils for that period are from the Sima de Los Huesos site (SH, Spain) and are associated with Neandertal-related individuals^47–50^. Nearly 500-foot bones have been discovered at SH. The foot fossils show a robust overall morphology with large facet joints, recalling those of the Late Pleistocene Neandertals^51^. However, still, they show some differences from Late Pleistocene *Homo* species, such as a particularly projected sustentaculum tali for calcanei, a vertically short body, broad fibular facets, and a mediolaterally broad talar head for tali that make this a distinct morphology for this Middle Pleistocene population^50,51^. However, these features are probably too tenuous to discern from the morphology of footprints compared to Late Pleistocene Neandertal populations.

On the contrary, the base of the Sima de los Huesos metatarsals is wider than that of the Neandertals, indicating a wider midfoot^50^. Such a difference might be detectable from the footprints. If the individuals who made the Matalascañas footprints were morphologically close to those of SH, their best-preserved footprints could be wider than Neandertal and *Homo sapiens* footprints left in similar substrate conditions.

The foot fossil record of other Middle Pleistocene European with chronologies similar to SH is not abundant. It is restricted to the fragmentary second metatarsal discovered in Arago (France) within abundant skeletal material associated with *Homo heidelbergensis* or *Homo erectus tautavelensis*^52^. This isolated second metatarsal described as not very robust^53^ does not allow for inference of the foot's morphology and, therefore, the resulting footprints. More recent (MIS 10-6) published remains comprise the second metatarsal from Sedia del Diavolo (MIS 8^54^) and a talus from Grotta del Poggio (MIS 7-6^55^), which are insufficient to ascertain the potential morphology of the footprint of the hominins of these chronologies.

Therefore, the results from^50,51^ show slight differences in the foot morphology within the Neandertal lineage. However, the scarce Middle Pleistocene fossil record for this anatomical region currently does not allow us to know when the “SH foot” became a Late Pleistocene “Neandertal foot” and whether these slight morphological differences would result in ichnological differences is still to be proven.

Therefore, taxonomic attribution is based only on chronological context, as is the case for most hominin footprints. The Middle Pleistocene European hominin fossils belong to the Neandertal lineage, either Neandertals or *Homo heidelbergensis s.l*. Therefore, the most likely taxonomic assignment for the Matalascañas footprints would be one of the taxa within this lineage. However, a more precise attribution seems complicated as there are many debates about the evolution of this lineage but also about the taxonomic definition of *Homo heidelbergensis*. Different models have been proposed for the evolution of the Neandertal lineage (e.g.,^56^ and references therein). This matter is still far from being solved, given the paucity of the fossil record and the new, more complicated evolutionary picture provided by the latest ancient DNA studies (e.g.,^49^). Based on the craniodental features, the pre-MIS 10 fossil record shows a great hominin morphological diversity with some demes (such as SH) morphologically and genetically linked to Neandertals^47–49^, while others (such as Aroeira, Ceprano or Mauer) show more primitive features^38,57,58^.

Additionally, it is possible to ascertain that not all anatomical features evolved at the same rate and that the Middle Pleistocene fossil record probably showed polymorphisms in different features with different percentages of appearance^47,48,59^. For example, despite its small size, the SH dentition is very Neandertal like^60,61^, but this deme shows a primitive occipital morphology and different morphotypes in the maxillary and radial morphology. The post-MIS 10 European fossil record shows, in general, an increasingly more “classic Neandertal” like morphology (e.g., Biache-Saint-Vaast^62^ but see^63^). Some of the MIS 7 fossil remains (such as Ehringsdorf) are regularly referred to as old Neandertals, but some fossil remains, such as the Montmaurin mandible^64^ and the Payre mandible^65^, document the persistence of plesiomorphic features even for these more recent periods. The latter could also reflect the aforementioned mosaic pattern of evolution in which some anatomical regions evolve at different rates.

In summary, based on the current knowledge of the human fossil record in Western Europe and the Iberian Peninsula in particular, and the new dating for the site, we consider the attribution of the Matalascañas footprints to individuals from the Neandertal lineage to be the most parsimonious decision. However, better-preserved footprints, in which Neandertal features such as a relatively wide foot, were noticeable would be necessary to test this hypothesis. On the other hand, studying the footprints can provide important palaeobiological information. Indeed, the first study of these footprints revealed that they were made by at least three individuals, including one child aged 6–8 years, probably seeking or bringing back resources, the orientation of these footprints towards animal tracks could indicate hunting behaviour^6^ a communal activity also detected in level TD10 of Gran Dolina, Atapuerca^66^. Furthermore, a child's participation in subsistence strategies could provide unique information about the distribution of activities within hominin groups and the cognitive learning of these activities. Indeed, although more and more archaeological discoveries have documented the complexity of subsistence strategies during the EMPH^67,68^, younger individuals' roles (learning, active participation) are currently unknown. Knowledge of these roles would provide unique information about these Middle Pleistocene hominins’ cultural and social behavior.

## Conclusions

Optically stimulated luminescence dating of four sedimentary levels at El Asperillo Clift establishes the age of the palaeosol PS1, which preserves hominin footprints, in 295.8 ± 17.8 ka (Middle Pleistocene). This new age is ~200 ka older than that established in a previous study for the aeolian unit U1 (106 ± 19 ka) located just above the PS1. Unit U1 is also analysed in this work with two samples from different sedimentary bodies yielding 274.0 ± 15.8 ka and 232.8 ± 13.1 ka. The youngest level studied is located at the base of the aeolian unit U2, with an age of 203.8 ± 12.7 ka. This new chronological framework implies changes in the palaeoenvironmental framework and a taxonomic reconsideration of these footprints. They were impressed in the last phases of MIS 9 in transition to MIS 8, a period of variations in the landscape characterised by an extensive coastal plain with large dune systems in an interglacial progressively extended by several tens of kilometres due to a sea retreated during the glacial period. We hypothesise that the hominin footprints were probably impressed by individuals from the Neandertal lineage, but better-preserved footprints with clear features related to the Neandertal lineage, such as a wide foot, would be necessary to prove this hypothesis. Nonetheless, the Matalascañas footprints complete a very partial hominin fossil record for the European Middle Pleistocene, especially concerning the small number of sites that have yielded hominin footprints.

## Supplementary Information

Supplementary Information.

## Data Availability

All data generated or analysed during this study are included in this published article [and its supplementary information files].
