# Supplementary material for: New dating of the Matalascañas footprints provides new evidence of the Middle Pleistocene (MIS 9-8) hominin paleoecology in southern Europe
Source: Sci Rep. 2022 Oct 19;12:17505. doi: 10.1038/s41598-022-22524-2 (PMC9581921; doi:10.1038/s41598-022-22524-2)
Supplement: Supplementary file 1 — Supplementary Information. [file 41598_2022_22524_MOESM1_ESM.docx]

**Supplementary Information:**

New dating of the Matalascañas footprints provides new evidence of the Middle Pleistocene (MIS 9-8) hominin paleoecology in southern Europe

Eduardo Mayoral^1,2*^, Jérémy Duveau^3,4^, Ana Santos^5^, Antonio Rodríguez Ramírez^1,2^, Juan A. Morales^1,2^, Ricardo Díaz-Delgado^6^, Jorge Rivera-Silva^7^, Asier Gómez-Olivencia^8,9,10^ and Ignacio Díaz-Martínez^11,12^

^1^Departamento de Ciencias de la Tierra, Facultad de Ciencias Experimentales, Campus de el Carmen, Universidad de Huelva, Huelva, Spain

^2^CCTH - Centro de Investigación Científico Tecnológico, Universidad de Huelva, Huelva, Spain

^3^DFG Center of Advanced Studies ‘Words, Bones, Genes, Tools’, Eberhard Karls University of Tübingen, Rümelinstrasse 23, D-72070 Tübingen, Germany.

^4^UMR 7194 Histoire Naturelle de l’Homme Préhistorique, CNRS, Muséum National d’Histoire Naturelle, Université Perpignan Via Domitia, Paris, France

^5^Departamento de Geología, Facultad de Geología, Campus de Llamaquique, Universidad de Oviedo, Oviedo, Spain.

^6^Estación Biológica de Doñana-CSIC, Sevilla, Spain

^7^Centro de Investigación, Tecnología e Innovación (CITIUS), Universidad de Sevilla, Sevilla, Spain

^8^Dept. Geología, Facultad de Ciencia y Tecnología, Universidad del País Vasco/Euskal Herriko Unibertsitatea, UPV/EHU. Barrio Sarriena s/n, 48940 Leioa, Spain.

^9^Sociedad de Ciencias Aranzadi. Zorroagagaina 11, 20014 Donostia-San Sebastián, Spain.

^10^Centro Mixto UCM-ISCIII de Investigación sobre Evolución y Comportamiento Humanos. Avda. Monforte de Lemos 5 (Pabellón 14), 28029 Madrid, Spain

^11^Universidad Nacional de Río Negro-IIPG. General Roca. Río Negro. Argentina

^12^Instituto de Investigación en Paleobiología y Geología (IIPG). CONICET. General Roca. Río Negro, Argentina

*Email: [mayoral@dgeo.uhu.es](mailto:mayoral@dgeo.uhu.es)

**Annex S1:** Dose-response curves and decay curves of the natural OSL of sample M1.

**Annex S2:** Dose-response curves and decay curves of the natural OSL of sample M2-3.

**Annex S3:** Dose-response curves and decay curves of the natural OSL of sample M2-2.

**Annex S4**: dose responses curves and decay curves of the natural OSL of sample M3.

**Table S1.** Selected European Middle Pleistocene sites with osteological remains.

| **Site** | **Level** | **Region** | **Fossil record** | **Chronology** | **Reference(s)** |
| --- | --- | --- | --- | --- | --- |

| Venosa |  | Basilicata, Italy | Femur portion | 640 ± 40 ka | MIS 16 | Lefèvre et al., 2010 |
| --- | --- | --- | --- | --- | --- | --- |
| Mauer | “Lower sands” | Mauer, Bade-Wurtemberg, Germany | 1 complete mandible | 609 ± 40 ka | MIS 15 | Schoetensack, 1908, Wagner et al., 2010 |
| Isernia la Pineta |  | Molise, Italy | Deciduous incisor | 583-561 ka | End of MIS 15 | Peretto et al., 2015 |
| Caune de l'Arago | Stratigraphic complex I, II and III | Tautavel, Pyrénées-Orientales, France | More than 150 dental and skeletal remains attributed to at least 30 individuals | 550-400 ka | MIS 14-MIS 11 | De Lumley, 1971, 2015; Falguères et al., 2015 |
| Boxgrove | 4/3M, 4u and 8ac | Sussex, United Kingdom | 2 lower incisors and 1 fragmented tibia | ≈ 500 ka | MIS 13 | Roberts et al., 1994; Streeter et al., 2001; Hillson et al., 2010 |
| Visogliano | Level 44 (2 of the human remains) | Friuli-Venezia Giulia, Italy | A right mandibular fragment, 5 isolated dental remains and three likely human tooth fragments | 480-440 ka | MIS 12 | Zanolli et al., 2018 |
| Fontana Ranuccio |  | Latium, Italy | 4 teeth | c. 450 ka | MIS 12 | Zanolli et al., 2018 and references therein |
| Galería |  | Sierra de Atapuerca, Burgos, Spain | A parietal fragment and a mandibular fragment | c. 450-250 ka | MIS 12-MIS 8 | Bermúdez de Castro and Rosas, 1992; Arsuaga et al., 1999; Berger et al., 2008 |
| Aroeira | Unit 2 | Almonda karst system, Portugal | 2 isolated teeth and a cranium | 436-390 ka | MIS 12-11 | Trinkaus et al., 2003; Daura et al., 2017 |
| Ceprano |  | Latium, Italy | Calvarium | 430-385 ka | MIS 12-MIS 11 | Manzi et al., 2010; Di Vincenzo et al., 2017 |
| Sima de los Huesos | LU6 | Sierra de Atapuerca, Burgos, Spain | > 7000 fossil remains; complete skeletons | 430 ka | End of MIS 12 | Arsuaga et al., 1997, 2014, 2015 and references therein |
| Petralona |  | Chalkidiki, Macedonia, Greece | A cranium |  | MIS 12? | Stringer, 1974, 1983; Stringer et al., 1979; Santos et al., 2014 and references therein |
| Mala Balanica | Level 3B | Niš, Serbia | Mandibular fragment |  | MIS 11 or older | Roksandic et al., 2011; Rink et al., 2013 |
| Bilzingsleben |  |  | 28 cranial fragments, 8 teeth and 1 mandibular fragment | 420-350 ka | MIS 11 | Vlček, 1978; Schwarcz et al., 1988; Vlček, et al., 2000; Müller and Pasda, 2011 and references therein |
| Pofi (Cava Pompi) |  | Latium, Italy | Portions of ulna and tibia, cranial fragment | 408-375 ka | MIS 11 | Passarello and Palmieri, 1968; Manzi et al., 2011; Pereira et al., 2018 |
| Swanscombe | IIb | Kent, United Kingdom | 3 fragments of a skull (occipital and parietals) | ≈ 400 ka | MIS11 | Wymer, 1955; Stringer and Hublin, 1999 |
| Reilingen | ? | Bade-Wurtemberg, Germany | 1 fragmented skull (right temporal, parietals, occipital) |  | MIS 11 - MIS 9 (?) | Czarnetzki, 1989; Dean et al., 1998 |
| Polledrara di Cecanibbio |  | Latium, Italy | Deciduous upper molar | 325 ± 6 ka - 304 ± 25 ka | MIS 9 | Anzidei et al., 2012; Pereira et al., 2017 |
| Steinheim |  | Steinheim, Bade-Wurtemberg, Germany | A nearly complete but deformed and crushed skull |  | MIS 9 | Berckhemer, 1936; Hublin, 1988 |
| Castel di Guido |  | Latium, Italy | 5 cranial remains and 2 femoral shafts |  | MIS 9 | Mariani-Costantini et al., 2001 and references therein |
| Sedia del Diavolo |  | Latium, Italy | Femoral shaft, second metatarsal | 295-290 ka | MIS 8 | Mallegni, 1986; Palombo et al., 2003-2004; Marra et al., 2017 |
| Ponte Mammolo |  | Latium, Italy | Femoral shaft |  | MIS 8 | Biddittu et al., 1987; Rubini et al., 1999; Marra et al., 2017 |
| Velika Balanica | Level 3a | Serbia | 4 fossil remains | 295 ± 74 ka and 285 ± 34 ka | MIS 8 | Roksandic et al., 2022 |
| Orgnac 3 | AL 6, AL 5b, AL 5a | Ardèche, France | 7 isolated teeth | 280-270 ka | MIS 8 | De Lumley, 1981; Bahain et al., 2022 |
| Lezetxiki | Level VIII | Arrasate, Basque Country, Spain | Humerus | > 164 +/- 9 ka | MIS 8/7 (context) | Basabe, 1966; de-la-Rúa et al., 2016 |
| Payre | E, F, Ga, Gb | Ardèche, France | A partial mandible including two molars and one premolar, a parietal fragment and thirteen isolated teeth |  | MIS 8-MIS 7 | Moncel et Condemi, 2007; Valladas et al., 2008; Verna et al., 2020 |
| Casal de’ Pazzi |  | Latium, Italy | Parietal fragment | 250-200 ka | MIS 7 | Manzi et al., 2001; Marra et al., 2017 |
| Grotta del Principe |  | Liguria, Italy | Coxal bone | 250-175 ka | MIS 7 -MIS 6 | de Lumley, 1972; Arellano et al., 2009 |
| Saccopastore |  | Latium, Italy | 2 crania | 245-220 ka | MIS 7 | Sergi, 1929; Breuil et Blanc, 1936; Marra et al., 2017 |
| Biache-Saint-Vaast (France) | IIA | Pas-de-Calais, France | 2 fragmented skulls | 236 ± 18 ka | MIS 7 | Tuffreau, 1978; Rougier, 2003; Guipert et al., 2011; Bahain et al., 2015 |
| Pontnewydd |  | Wales, United Kingdom | 17 teeth and a fragmented jaw | ≈ 225 ka | MIS 7 | Green et al., 1981; Compton and Stringer, 2015 |
| Mollet | Layer 5 | Serinyà, Girona, Catalonia, Spain | Upper first right molar | c. 215 ka | MIS 7 | Maroto et al., 2012 |
| Kozarnika | Layer 10b | Bulgaria | Oblast de Gabrovo, Bulgaria | 201 ± 17 ka | MIS 7 | Tillier et al., 2017; Heydari et al., 2022 |
| Cueva del Bolomor | Level XIII | Tavernes de la Valldigna, València, Spain | Right upper canine |  | MIS 7 | Arsuaga et al., 2012 |
| Weimar-Ehringsdorf | Unterer Travertin | Thuringia, Germany | 32 cranial and postcranial remains and a block with 5 vertebrae and 13 ribs |  | MIS 7 | Vlček, 1993; Mallick and Frank, 2002 |
| Montmaurin-La Niche Cave | C3 | Haute-Garonne, France | A mandible including its right and left molar series, a cervical vertebra and an incomplete tibia |  | MIS 7 | Vallois, 1955; Cammas et Tavoso, 1986; Vialet et al., 2018; de Pinillos et al., 2020 |
| Apidima |  | Greece | 2 crania | c. 210 ka (Apidima 1); c. 170 ka (Apidima 2) | MIS 7 -MIS 6 | Harvati et al., 2019 |
| Grotta del Poggio |  | Campania, Liguria | 1 molar, 1 talus |  | MIS 7- MIS 6 | Boscato et al., 2009; Manzi et al., 2011 and references therein |
| Lazaret | CI, CII, CIII | Alpes-Maritimes, France | 28 cranial  (frontal, parietal, teeth...) and postcranial  (humerus, femurs...) remains | 190-120 ka | MIS 6 | De Lumley, 1973, 2018 |
| Altamura |  | Puglia, Italy | Skeleton | 172 ± 15 ka - 130.1 ± 1.9 ka | MIS 6 | Lari et al., 2015 |
| Combe Grenal | Couche 60 | Domme, France | Lower right decidual central incisor |  | MIS 6 | Maureille et al., 2009-2010 |
| Krapina | Present in at least 7 of the 9 levels. Significant presence in level 4 | Croatia | Several hundred remains including all anatomical parts (MNI=24). | 130 ka | MIS 5e | Trinkaus, 1975; Radovčić and Wolpoff, 1988; Rink et al., 1995; White & Toth (pers. comm.) in Bocquet-Appel and Arsuaga, 1999 |
| Vértesszőllős |  | Komárom-Esztergom, Hungary | An occipital bone and 2 deciduous teeth | Mid-part of Middle Pleistocene |  | Thoma, 1969, 1978 and references therein |
| Marathousa 1 | Unknown (intrusive?) | Megalopolis, Greece | A single upper third molar | late Lower Pleistocene- early Middle Pleistocene |  | Sickenberg, 1975; Panagopoulou et al., 2015; Harvati, 2016 |

**Table S2.** European Middle Pleistocene sites with ichnological records.

| **Site** | **Level** | **Region** | **Fossil record** | **Chronology** | **References(s)** |  |
| --- | --- | --- | --- | --- | --- | --- |
| Terra Amata | C16 | Mont Boron,  Nice, Alpes-Maritimes, France | A single footprint that reflects a slide of the foot | 380 ± 80 ka  MIS 11 | De Lumley et al., 2011 | |
| Roccamonfina | LS07 | Campania, Italy | At least 81 footprints distributed in 4 trackways | 349 ± 3 ka  MIS 10 | Mietto et al., 2003; Scaillet et al., 2008; Panarello et al., 2020 | |
| Matalascañas | HTS | Doñana shoreline, Spain | 87 footprints | 295 ± 17 ka  MIS 9-8 | Mayoral et al., 2021; This study | |
| Biache-Saint Vaast | IIA | Pas-de-Calais, France | 1 potential footprint (poorly preserved) | 236 ± 18 ka  MIS 7 | Tuffreau, 1978; Bahain et al., 2015 | |
| Theopetra Cave | II2 | Thessaly, Greece | 4 footprints made by children | 135 ka  MIS 6 | Manolis et al., 2000; Kyparissi-Apostolika and Manolis, 2021 | |

**References**

Anzidei, A. P., Bulgarelli, G. M., Catalano, P., Cerilli, E., Gallotti, R., Lemorini, C., Milli, S., Palombo, M. R., Pantano, W. & Santucci, E. Ongoing research at the late Middle Pleistocene site of La Polledrara di Cecanibbio (central Italy), with emphasis on human–elephant relationships. *Quat.* *Inter*. **255**, 171–187 (2012).

Arellano, A. Les grands mammifères des niveaux moustériens de l’Abri Mochi (grottes de Grimaldi, Vintimille, Italie). Fouilles de 1949. *Bulletin du musée d’Anthropologie préhistorique de Monaco* **49**, 29–39 (2009).

Arsuaga, J. L., Martínez, I., Gracia, A. & Lorenzo, C. The Sima de los Huesos crania (Sierra de Atapuerca, Spain). A comparative study. *J.* *Hum.* *Evol.* **33**, 219–281 (1997).

Arsuaga, J. L., Gracia, A., Lorenzo, C., Martínez, I. & Pérez, P. J. *Resto craneal humano de Galería/Cueva de los Zarpazos (Sierra de Atapuerca)*. in Atapuerca: ocupaciones humanas y paleoecología del yacimiento de Galería. (eds. Carbonell, E., Rosas, A. & Díez, J. C.) 233–235 (Junta de Castilla y León, Valladolid 1999).

Arsuaga, J. L., Fernández Peris, J., Gracia-Téllez, A., Quam, R., Carretero, J. M., Barciela González, V., Blasco, R., Cuartero, F. & Sañudo, P. Fossil human remains from Bolomor Cave (Valencia, Spain). *J.* *Hum*. *Evol*. **62**, 629-639 (2012).

Arsuaga, J. L., Martínez, I., Arnold, L. J., Aranburu, A., Gracia-Téllez, A., Sharp, W. D., Quam, R. M., Falguères, C., Pantoja-Pérez, A., Bischoff, J., Poza-Rey, E., Parés, J. M., Carretero, J. M., Demuro, M., Lorenzo, C., Sala, N., Martinón-Torres, M., García, N., Alcázar de Velasco, A., Cuenca-Bescós, G., Gómez-Olivencia, A., Moreno, D., Pablos, A., Shen, C.-C., Rodríguez, L., Ortega, A. I., García, R., Bonmatí, A., Bermúdez de Castro, J. M. & Carbonell, E. Neandertal roots: Cranial and chronological evidence from Sima de los Huesos. *Science* **344**, 1358–1363 (2014).

Arsuaga, J. L., Carretero, J.-M., Lorenzo, C., Gómez-Olivencia, A., Pablos, A., Rodríguez, L., García-González, R., Bonmatí, A., Quam, R. M., Pantoja-Pérez, A., Martínez, I., Aranburu, A., Gracia-Téllez, A., Poza-Rey, E., Sala, N., García, N., Alcázar de Velasco, A., Cuenca-Bescós, G., Bermúdez de Castro, J. M. & Carbonell, E. Postcranial morphology of the middle Pleistocene humans from Sima de los Huesos, Spain. *PNAS* **112**, 11524–11529 (2015).

Bahain, J. J., Falgueres, C., Laurent, M., Dolo, J. M., Shao, Q., Auguste, P. & Tuffreau, A. ESR/U-series dating of faunal remains from the paleoanthropological site of Biache-Saint-Vaast (Pas-de-Calais, France). *Quat*. *Geochronology* **30**, 541–546 (2015).

Bahain, J. J., Mercier, N., Valladas, H., Falguères, C., Masaoudi, H., Joron, J. L., Froget, L., Moigne, A-M., Combier, J. & Moncel, M. H. Reappraisal of the chronology of Orgnac 3 Lower-to-Middle Paleolithic site (Ardèche, France), a regional key sequence for the Middle Pleistocene of southern France. *J. Hum. Evol.* **162**, 103092 (2022).

Basabe, J. M. El húmero premusteriense de Lezetxiki (Guipúzcoa). *Munibe* **18**, 13–32 (1966).

Berckhemer, F. Der Urmenschenschädel aus den zwischeneiszeitlichen Fluss-Schottern von Steinheim an der Murr. Württ. Naturaliensammlung (1936).

Berger, G. W., Pérez-González, A., Carbonell, E., Arsuaga, J. L., Bermúdez de Castro, J. M. & Ku, T. L. Luminescence chronology of cave sediments at the Atapuerca paleoanthropological site, Spain. *J*. *Hum*. *Evol*. **55**, 300–311 (2008).

Bermúdez de Castro, J. M. & Rosas, A. A human mandibular fragment from the Atapuerca Trench (Burgos, Spain). *J*. *Hum*. *Evol*. **22**, 41–46 (1992).

Biddittu, I., Mallegni, F. & Segre, A. G. Riss age human remain, recovered from pleistocene deposits in Ponte Mammolo (Rome-Italy). *Zeitschrift für Morphologie und Anthropologie* **77**, 181–191 (1987).

Bocquet-Appel, J. P. & Arsuaga, J. L. Age Distributions of Hominid Samples at Atapuerca (SH) and Krapina Could Indicate Accumulation by Catastrophe. *J*. *Archaeol*. *Sci*. **26**, 327–338 (1999).

Boscato, P., Boschian, G., Caramia, F. & Gambassini, P. Il Riparo del Poggio a Marina di Camerota (Salerno): culture ed ambiente. *Rivista di Scienze Preistoriche* **59**, 5–40 (2009).

Breuil, H. & Blanc, A. C. Le nouveau crane de Saccopastore, Rome. *L’Anthropologie* **46**, 1–16 (1936).

Cammas, R. & Tavoso, A. Nouveaux restes humains issus du remplissage de la Niche (Montmaurin, Haute-Garonne). *Comptes rendus de l'Académie des sciences Série 2* **302**, 609–614 (1986).

Compton, T. & Stringer, C. The morphological affinities of the Middle Pleistocene hominin teeth from Pontnewydd Cave, Wales. *J. Quat. Sci.* **30**, 713–730 (2015).

Czarnetzki, A. Ein archaischer Hominidencalvariarest aus einer Kiesgrube in Reilingen, Rhein-Neckar-Kreis. *Quartär–Internationales Jahrbuch zur Erforschung des Eiszeitalters und der Steinzeit*, 191–201 (1989).

Daura, J., Sanz, M., Arsuaga, J. L., Hoffmann, D. L., Quam, R. M., Ortega, M. C., Santos, E., Gómez, S., Rubio, A., Villaescusa, L., Souto, P., Mauricio, J., Rodrigues, F., Ferreira, A., Godinho, P., Trinkaus, E. & Zilhão, J. New Middle Pleistocene hominin cranium from Gruta da Aroeira (Portugal). *PNAS* **114**, 3397–3402 (2017).

Dean, D., Hublin, J. J., Holloway, R. & Ziegler, R. On the phylogenetic position of the pre-Neandertal specimen from Reilingen, Germany. *J*. *Hum*. *Evol*. **34**, 485–508 (1998).

De Pinillos, M. M., Martín-Francés, L., de Castro, J. M. B., García-Campos, C., Modesto-Mata, M., Martinón-Torres, M. & Vialet, A. Inner morphological and metric characterization of the molar remains from the Montmaurin-La Niche mandible: The Neanderthal signal. *J*. *Hum*. *Evol*. **145**, 102739 (2020).

De-la-Rúa, C., Altuna, J., Hervella, M., Kinsley, L. & Grün, R. Direct U-series analysis of the Lezetxiki humerus reveals a Middle Pleistocene age for human remains in the Basque Country (northern Iberia). *J. Hum. Evol.* **93**, 109–119 (2016).

De Lumley, H. Découverte de restes humains anténéandertaliens datés du début du Riss à la caune de l’Arago (Tautavel, Pyrénées-Orientales). *Comptes Rendus l’Académie des Sci Paris* **500**, 1729–1742 (1971).

De Lumley, M. -A. L'Os iliaque anténéandertalien de la Grotte du Prince (Grimaldi, Ligurie italienne). *Bulletin du Musée d'Anthropologie Préhistorique de Monaco* **18**, 89–112 (1972).

De Lumley, M. -A. Anténéandertaliens et néandertaliens du bassin méditerranéen occidental européen. *Études Quaternaires* **2**, 626 (1973).

De Lumley, M. A. *Les restes humains d’Orgnac 3*. in Les premiers habitants de l’Europe (1 500 000–100 000 ans)., 143–145 (*Laboratoire de Préhistoire du Musée de l’Homme*, Paris, 1981).

De Lumley, M. -A., Lamy, P. & Mafart, B. *Une empreinte de pied humain acheuléen dans la dune littorale du site de Terra Amata. Ensemble stratigraphique C1b*. in Terra Amata: Nice, Alpes-Maritimes, France. (ed. de Lumley, H.), Tome II, Palynologie, anthracologie, faunes, mollusques, écologie et biogéomorphologie, paléoanthropologie, empreinte de pied humain, coprolithes **2**, 483–507, (CNRS Editions, Paris, 2011).

De Lumley, M. A. L’homme de Tautavel. Un *Homo erectus* européen évolué. *Homo erectus tautavelensis*. *l'Anthropologie* ***119***, 303–348 (2015).

De Lumley, M. -A. *Les restes humains fossiles de la grotte du Lazaret. Nice, Alpes-Maritimes, France. Des Homo erectus européens évolués en voie de néandertalisation* (pp. 664). (CNRS éditions, 2018).

Di Vincenzo, F., Profico, A., Bernardini, F., Cerroni, V., Dreossi, D., Schlager, S., Zaio, P., Benazzi, S., Biddittu, I., Rubini, M., Tuniz, C. & Manzi, G. Digital reconstruction of the Ceprano calvarium (Italy), and implications for its interpretation. *Sci. Rep.* **7**, 13974 (2017).

Falguères, C., Shao, Q., Han, F., Bahain, J. J., Richard, M., Perrenoud, C. & Moigne, A. M. New ESR and U-series dating at Caune de l'Arago, France: A key-site for European Middle Pleistocene. *Quat. Geochronology* **30**, 547–553 (2015).

Green, H. S., Stringer, C. B., Collcutt, S. N., Currant, A. P., Huxtable, J., Schwarcz, H. P., Debenham, N., Embleton, C., Bull, P., Molleson, T. I. & Bevins, R. E. (1981). Pontnewydd Cave in Wales—a new Middle Pleistocene hominid site. *Nature*, **294**(5843), 707–713 (1981).

Guipert, G., de Lumley, M. A., Tuffreau, A. & Mafart, B. A late Middle Pleistocene hominid: Biache-Saint-Vaast 2, north France. *C. R. Palevol*. **10**, 21–33 (2011).

Harvati, K. Paleoanthropology in Greece: recent findings and interpretations. *Paleoanthr. Balkans and Anatolia*, 3–14 (2016).

Harvati, K., Röding, C., Bosman, A. M., Karakostis, F. A., Grün, R., Stringer, C., Karkanas, P., Thompson, N. C., Koutoulidis, V., Moulopoulos, L. A., Gorgoulis, V. G. & Kouloukoussa, M. Apidima Cave fossils provide earliest evidence of *Homo sapiens* in Eurasia. *Nature* **571**, 500–504 (2019).

Heydari, M., Guérin, G., Sirakov, N., Fernandez, P., Ferrier, C., Guadelli, A., Leblanc, J.-C., Taneva, S., Sirakova, S., Guadelli, J.-L.. The last 30,000 to 700,000 years ago: Unravelling the timing of human settlement for the Palaeolithic site of Kozarnika. Quat Sci Rev 291, 107645 (2022).

Hillson, S. W., Parfitt, S. A., Bello, S. M., Roberts, M. B. & Stringer, C. B. Two hominin incisor teeth from the middle Pleistocene site of Boxgrove, Sussex, England. *J. Hum. Evol.* **59**, 493–503 (2010).

Hublin, J. J. Les plus anciens représentants de la lignée prénéandertalienne. L’homme de Néandertal 3, l (1988).

Kyparissi-Apostolika, N. & Manolis, S. K. *Reconsideration of the Antiquity of the Middle Palaeolithic Footprints from Theopetra Cave* *(Thessaly, Greece)*. in Reading Prehistoric Human Tracks. (eds. Pastoors, A. & Lenssen-Er, T.), 169–182, (Springer International Publishing, Cham, 2021).

Lari, M., Di Vincenzo, F., Borsato, A., Ghirotto, S., Micheli, M., Balsamo, C., Collina, C., De Bellis, G., Frisia, S., Giacobini, G., Gigli, E., Hellstrom, J.C., Lannino, A., Modi, A., Pietrelli, A., Pilli, E., Profico, A., Ramirez, O., Rizzi, E., Vai, S., Venturo, D., Piperno, M., Lalueza-Fox, C., Barbujani, G., Caramelli, D. & Manzi, G. The Neanderthal in the karst: First dating, morphometric, and paleogenetic data on the fossil skeleton from Altamura (Italy). *J. Hum. Evol.* **82**, 88–94 (2015).

Lefèvre, D., Raynal, J.-P., Vernet, G., Kieffer, G. & Piperno, M. Tephro-stratigraphy and the age of ancient southern Italian Acheulean settlements: the sites of loreto and Notarchirico (Venosa, Basilicata, Italy). *Quat. Int*. 223e224, 360e368 (2010).

Mallegni, F. Les restes humains du gisement de Sedia del Diavolo (Rome) remontant au Riss final. *L'Anthropologie* **90**, 539–553 (1986).

Mallick, R. & Frank, N. A new technique for precise uranium-series dating of travertine micro-samples. *Geochim*. *Cosmochim*. *Acta* **66**, 4261–4272 (2002).

Manolis, S., Aiello, L., Henessy, R., & Kyparissi-Apostolika, N. Middle Palaeolithic footprints from Theopetra cave (Thessaly, Greece). Greek Ministry of Culture and Institute for Aegean Prehistory (2000).

Manzi, G., Palombo, M. R., Caloi, L. & Mallegni, F. Transitions in human evolution and faunal changes during the Pleistocene in Latium (Central Italy). In *Proceedings of the 1st International Congress, The World of Elephants, Roma, CNR-Roma* 59–66, (2001).

Manzi, G., Magri, D., Milli, S., Palombo, M. R., Margari, V., Celiberti, V., Barbieri, M., Barbieri, M., Melis, R. T., Rubini, M., Ruffo, M., Saracino, B., Tzedakis, P. C., Zarattini, A., Biddittu, I. The new chronology of the Ceprano calvarium (Italy). *J. Hum. Evol.* **59**, 580–585 (2010).

Manzi, G., Magri, D. & Palombo, M. R. Early–Middle Pleistocene environmental changes and human evolution in the Italian peninsula. *Quat*. *Sci*. *Rev*. **30**, 1420–1438 (2011).

Mariani-Costantini, R., Ottini, L., Caramiello, S., Palmirotta, R., Mallegni, F., Rossi, A., Frati, L. & Capasso, L. Taphonomy of the fossil hominid bones from the Acheulean site of Castel di Guido near Rome, Italy. *J. Hum. Evol.* **41**, 211–225 (2001).

Maureille, B., Garralda, M. D., Madelaine, S., Turq, A. & Vandermeersch, B. Le plus ancien enfant d'Aquitaine: Combe-Grenal 31 (Domme, France). *Paleo* **21**, 189–202 (2009-2010).

Maroto, J., Julià, R., López-García, J. M. & Blain, H.-A. Chronological and environmental context of the Middle Pleistocene human tooth from Mollet Cave (Serinyà, NE Iberian Peninsula). *J. Hum. Evol.* **62**, 655–663 (2012).

Marra, F., Ceruleo, P., Pandolfi, L., Petronio, C., Rolfo, M. F. & Salari, L. The Aggradational Successions of the Aniene River Valley in Rome: Age Constraints to Early Neanderthal Presence in Europe. *PLoS One* **12**, e0170434 (2017).

Mayoral, E., Díaz-Martínez, I., Duveau, J., Santos, A., Ramírez, A. R., Morales, J. A., Morales, L. A., & Díaz-Delgado, R. Tracking late Pleistocene Neandertals on the Iberian coast. *Sci. Rep.*, *11*(1), 1–12 (2021).

Mietto, P., Avanzini, M. & Rolandi, G. Human footprints in Pleistocene volcanic ash. *Nature* **422**(6928), 133–133 (2003).

Moncel, M. H. & Condemi, S. The human remains of the site of Payre (SE France, MIS 7-5). remarks on stratigraphic position and interest. *Anthropologie* **45**, 19 (2007).

Müller, W. & Pasda, C. Site formation and faunal remains of the Middle Pleistocene site Bilzingsleben. *Quartär* **58**, 25–49 (2011).

Palombo, M. R., Milli, S. & Rosa, C. Remarks on the biochronology of the Late Middle Pleistocene mammalian faunal complexes of the Campagna Romana (Latium, Italy). *Geologica Romana* **37**, 135–143 (2003-2004).

Panagopoulou, E., Tourloukis, V., Thompson, N., Athanassiou, A., Tsartsidou, G., Konidaris, G. E., Giusti, D., Karkanas, P. & Harvati, K. Marathousa 1: A new Middle Pleistocene archaeological site from Greece. *Antiquity Project Gallery* **89** (343) (2015).

Panarello, A., Palombo, M. R., Bikdditti, I., Di Vito, M. A., Farinaro, G. & Mietto, P. On the devil's tracks: unexpected news from the Foresta ichnosite (Roccamonfina volcano, central Italy). *J. Quat. Scie*. **35**, 444–456 (2020).

Passarello, P &, Palmieri, A. Studio sui resti umani di tibia e di ulna provenienti da strati pleistocenici della Cava Pompi di Pofi (Frosinone). *Rivista di Antropologia* **55**, 139–162 (1968).

Pereira, A., Nomade, S., Falguères, C., Bahain, J.-J., Tombret, O., Garcia, T., Voinchet, P., Bulgarelli, G.-M. & Anzidei, A.-P. 40Ar/39Ar and ESR/U-series data for the La Polledrara di Cecanibbio archaeological site (Lazio, Italy). *J. Archaeol. Sci.* **15**, 20–29 (2017).

Pereira, A., Nomade, S., Moncel, M.-H., Voinchet, P., Bahain, J.-J., Biddittu, I., Falguères, C., Giaccio, B., Manzi, G., Parenti, F., Scardia, G., Scao, V., Sottili, G. & Vietti, A. Integrated geochronology of Acheulian sites from the southern Latium (central Italy): Insights on human-environment interaction and the technological innovations during the MIS 11-MIS 10 period. *Quat. Sci. Rev.* **187**, 112–129 (2018).

Peretto, C., Arnaud, J., Moggi-Cecchi, J., Manzi, G., Nomade, S., Pereira, A., Falguères, C., Bahain, J.-J., Grimaud-Hervé, D., Berto, C., Sala, B., Lembo, G., Muttillo, B., Gallotti, R., Thun Hohenstein, U., Vaccaro, C., Coltorti, M. & Arzarello, M. A Human Deciduous Tooth and New 40Ar/39Ar Dating Results from the Middle Pleistocene Archaeological Site of Isernia La Pineta, Southern Italy. *PloS One* 10, e0140091 (2015).

Radovčić, J. & Wolpoff, M. H. The Krapina hominids: an illustrated catalog of skeletal collection. Mladost (1988).

Rink, W. J., Schwarcz, H. P., Smith, F. H. & Radovĉiĉ, J. ESR ages for Krapina hominids. *Nature*, **378** (6552), 24–24 (1995).

Rink, W. J., Mercier, N., Mihailović, D., Morley, M. W., Thompson, J. W. & Roksandic, M. New Radiometric Ages for the BH-1 Hominin from Balanica (Serbia): Implications for Understanding the Role of the Balkans in Middle Pleistocene Human Evolution. *PloS One* 8, e54608 (2013).

Roberts, M. B., Stringer, C. B. & Parfitt, S. A. A hominid tibia from Middle Pleistocene sediments at Boxgrove, UK. *Nature* **369** (6478), 311–313 (1994).

Roksandić, M., Mihailović, D., Mercier, N., Dimitrijević, V., Morley, M. W., Rakočević, Z., Mihailović, B., Guibert, P. & Babb, J. A human mandible (BH-1) from the Pleistocene deposits of Mala Balanica cave (Sićevo Gorge, Niš, Serbia).  *J. Hum. Evol.* **61**, 186–196 (2011).

Roksandić, M., Radović, P., Lindal, J. & Mihailović, D. Early Neanderthals in contact: The Chibanian (Middle Pleistocene) hominin dentition from Velika Balanica Cave, Southern Serbia. *J. Hum. Evol.* **166**, 103175 (2022).

Rougier, H. Étude descriptive et comparative de Biache-Saint-Vaast 1 (Biache-Saint-Vaast, Pas-de-Calais, France). Ph.D. thesis, Université de Bordeaux I, Bordeaux (2003).

Rubini, M., Mogliazza, S., Bonafede, E. & Mallegni, F. New considerations on the Ponte Mammolo fossil femur (Middle Pleistocene, Rome, Italy). *Archivio per l'antropologia e la etnologia* **129**, 145–160 (1999).

Santos, E., Garcia, N., Carretero, J. M., Arsuaga, J. L. & Tsoukala, E. Endocranial traits of the Sima de los Huesos (Atapuerca, Spain) and Petralona (Chalkidiki, Greece) Middle Pleistocene ursids. Phylogenetic and biochronological implications. *Annales de Paléontologie* **100**, 297–309 (2014).

Scaillet, S., Vita-Scaillet, G. & Guillou, H. Oldest Human Footprints Dated by Ar/Ar. *Earth Planet. Sci. Lett*. **275**, 320–325 (2008).

Schoetensack, O. The mandible of *Homo heidelbergensis* from the sands of Mauer near Heidelberg—a contribution to the palaeontology of man. Leipzig: Engelmann. (1908).

Schwarcz, H. P., Grün, R., Latham, A. G., Mania, D. & Brunnacker, K. The Bilzingsleben archaeological site: new dating evidence. *Archaeometry* 30**,** 5–17 (1988).

Sergi, S. La scoperta di un cranio del tipo di Neanderthal presso Roma. *Riv. Antropol*. **28**, 457–462 (1929).

Sickenberg, O. Eine Säugertierfauna des tieferen Bihariums aus den Becken von Megalopolis (Peloponnes, Griechenland). *Annales Géologiques des Pays Helléniques* **27**, 26–71 (1975).

Streeter, M., Stout, S. D., Trinkaus, E., Stringer, C. B., Roberts, M. B. & Parfitt, S. A. Histomorphometric age assessment of the Boxgrove 1 tibial diaphysis. *J. pHum. Evol.* **40**, 331–338 (2001).

Stringer, C.B. A multivariate study of the Petralona skull. *J. Hum. Evol.* **3**, 397–404 (1974).

Stringer, C. B. & Hublin, J. J. New age estimates for the Swanscombe hominid, and their significance for human evolution. *J. Hum. Evol.* **6**, 873–877 (1999).

Stringer, C. B. Some further notes on the morphology and dating of the Petralona hominid. *J. Hum. Evol.* **12**, 731–742 (1983).

Stringer, C. B., Howell, F. C. & Melentis, J. K. The significance of the fossil hominid skull from Petralona, Greece. *J. Archaeol. Sci.* **6**, 235–253 (1979).

Thoma, A. Biometrische studie über das occipitale von Vértesszöllös. Zeitschrift für Morphologie und Anthropologie, 229–241 (1969).

Thoma, A. Some notes on Wolpoff's notes on the Vertesszollos occipital. *J. Hum. Evol.* **7**, 323–325 (1978).

Tillier, A.-M., Sirakov, N., Guadelli, A., Fernandez, P., Sirakova, S., Dimitrova, I., Ferrier, C., Guérin, G., Heidari, M., Krumov, I., Leblanc, J.-C., Miteva, V., Popov, V., Taneva, S., Guadelli, J.-L.. Evidence of Neanderthals in the Balkans: The infant radius from Kozarnika Cave (Bulgaria). J. Hum. Evol., 111, 54-62 (2017).

Trinkaus, E. The Neandertals from Krapina, northern Yugoslavia: an inventory of the lower limb remains. *Zeitschrift für Morphologie und Anthropologie*, 44–59 (1975).

Trinkaus, E., Marks, A. E., Brugal, J.-P., Bailey, S. E., Rink, W. J. & Richter, D. Later Middle Pleistocene human remains from the Almonda Karstic system, Torres Novas, Portugal. *J. Hum. Evol.* **45**, 219–226 (2003).

Tuffreau, A. Les fouilles du gisement paléolithique de Biache-Saint-Vaast (Pas-de-Calais): années 1976 et 1977-premiers résultats. *Quaternaire* **15**, 46–55 (1978).

Valladas, H., Mercier, N., Ayliffe, L. K., Falguères, C., Bahain, J. J., Dolo, J.-M., Froget, L., Joron, J.-L., Masaoudi, H., Reyss, J.-L. & Moncel, M-H. Radiometric dates for the middle Palaeolithic sequence of Payre (Ardeche, France). *Quat. Geochronology* **3**, 377–389 (2008).

Vallois, H. V. La mandibule humaine pré-moustérienne de Montmaurin. *Comptes rendus hebdomadaires des séances de l’Académie des sciences*, Paris, **240**, 1577–1579 (1955).

Verna, C., Détroit, F., Kupczik, K., Arnaud, J., Balzeau, A., Grimaud-Hervé, D., Bertrand, S., Riou, B. & Moncel, M. H. The Middle Pleistocene hominin mandible from Payre (Ardèche, France). *J. Hum. Evol.* **144**, 102775 (2020).

Vialet, A., Modesto-Mata, M., Martinón-Torres, M., Martínez de Pinillos, M. & Bermudez de Castro, J. M. A reassessment of the Montmaurin-La Niche mandible (Haute Garonne, France) in the context of European Pleistocene human evolution. PloS One **13**(1), e0189714 (2018).

Vlček, E. A new discovery of *Homo erectus* in Central Europe. *J. Hum. Evol.* **7**, 239–251 (1978).

Vlček, E. Fossile Menschenfunde von Weimar-Ehringsdorf. Theiss, Stuttgart (1993).

Vlček, E., Mania, D. & Mania, U. A new find of a Middle Pleistocene mandible from Bilzingsleben, Germany. *Naturwissenschaften* **87**, 264–265 (2000).

Wagner, G. A., Krbetschek, M., Degering, D., Bahain, J.-J., Shao, Q., Falguères, C., Voinchet, P., Dolo, J.-M., Garcia, T. & Rightmire, G. P. Radiometric dating of the type-site for *Homo heidelbergensis* at Mauer, Germany. PNAS **107**, 19726–19730 (2010).

Wymer, J. A further fragment of the Swanscombe skull. *Nature* **176**(4479), 426–427 (1955).

Zanolli, C., Martinón-Torres, M., Bernardini, F., Boschian, G., Coppa, A., Dreossi, D., Mancini, L., Martínez de Pinillos, M., Martín-Francés, L., Bermúdez de Castro, J.M., Tozzi, C., Tuniz, C., Macchiarelli, R., 2018. The Middle Pleistocene (MIS 12) human dental remains from Fontana Ranuccio (Latium) and Visogliano (Friuli-Venezia Giulia), Italy. A comparative high resolution endostructural assessment. Plos one 13, e0189773.
